# Development and optimisation of an intervention to increase the intention to act on health and health equity within the private sector of urban development: an evidence, theory and Person-Based Approach

**DOI:** 10.1186/s12889-025-22559-w

**Published:** 2025-04-26

**Authors:** Sophie L. Turnbull, Martha Jordan, Rebecca J. Linnett, Daniel Black, Harry Knibb, Zoe Sharpe, Krista Bondy

**Affiliations:** 1 University of Bristol, Bristol, UK; 2 University of Bath, Bath, UK; 3 University of Stirling, Stirling, UK; 4 Oxford Properties, London, UK; 5 Dandara Living, Bristol, UK

**Keywords:** Public health, Health equity, Psychosocial intervention, Chronic disease, Decision making

## Abstract

**Background:**

There is growing evidence that exposure to unhealthy urban environments increases the risk of developing non-communicable diseases (e.g. diabetes, cardiovascular disease, and respiratory illness), with marginalised communities bearing the greatest burden. However, to date, evidence alone has not been sufficient to make health a top priority in the development of urban environments.

**Methods:**

The aim of this study was to develop and optimise an intervention to increase the intention to act on health and health inequalities by private sector professionals working in urban development, with a focus on consultants and developers. The ‘Changing Mindsets’ intervention was developed through an iterative co-production process using the Person-Based Approach method, drawing on evidence and a novel theoretical framework.

**Results:**

Intervention development consisted of three stages. Stage 1 involved the collation of theory and evidence, which included the development of a novel theoretical framework, primary mixed methods research and stakeholder engagement. Stage 2 was the intervention modelling phase, where the findings from Stage 1 were integrated through the guiding principles and behavioural analysis tables, which informed the logic model. Stage 3 involved iterative intervention optimisation with members of the target population. The intervention was comprised of two elements: 1) An intervention session consisting of a presentation with group discussion presented by one of the two industry partners working in the private sector of urban development, and 2) A website signposting to tools and resources, networks to support prioritising and integrating health into urban development, and examples of how other organisations have done so.

**Conclusions:**

We have provided insights into how complex interdisciplinary theory can be combined with evidence of the target group’s needs, issues and challenges using established methodology from the Person-Based Approach and behavioural science. Changing Mindsets is currently being evaluated for its effectiveness and acceptability in the target population. Subsequent to this, there are plans to adapt the intervention to increase the intention to act on other social issues and for other populations.

**Trial registration:**

ISRCTN12310546 registered on the 30 th March 2021.

**Supplementary Information:**

The online version contains supplementary material available at 10.1186/s12889-025-22559-w.

## Background

Evidence is mounting regarding the link between the quality of the urban environment and the development of non-communicable diseases (NCDs) such as diabetes, cardiovascular disease, and respiratory illness (e.g. asthma and chronic obstructive pulmonary disease) [1–3]. The global burden of NCDs is rising rapidly, accounting for 74% of all global deaths [4]. NCDs disproportionately affect those from lower socio-economic groups who have fewer resources to leverage to protect themselves from developing NCDs, and to manage their condition when they are diagnosed [5, 6]. These resources include the environment within which they live, grow and work.

Many aspects of the built environment can affect health and health inequalities. For example, there is evidence that green space has a beneficial impact on physical and mental health, while lack of access to green spaces and infrastructure can contribute towards mental ill-health through reductions in social cohesion, air quality, visual stimulation and physical activity [7–9]. Exposure to higher levels of air pollutants – primarily from car emissions, but also brake particulates—can increase the risk of mortality from conditions such as cardiovascular disease and lung cancer [10, 11]. Poor quality housing is estimated to cost the NHS £1.4bn each year due to issues such as damp and mould [12]; air pollution causes 40,000 deaths in the UK per year and costs £20 billion [13]. Those living in more deprived areas experience greater exposure to health damaging environmental conditions, such as noise, pollution and heavy traffic pollution [14–16]. Whilst having less access to protective factors such as access to safe green spaces to play and exercise, healthy food stores and the best health-care facilities and clinicians [16]. However, to date, the compelling evidence regarding the link between illness and urban environments has been insufficient to increase the prioritisation of developing healthy environments within the urban development sector [17, 18].

The ‘Tackling the Root Causes Upstream of Unhealthy Urban Development’ (TRUUD) project is a £10 million transdisciplinary research consortium funded by the UK Prevention Research Partnership, aimed at addressing health and health equity within urban development. The TRUUD program has had two distinct phases. Phase 1, which ran from October 2019 to June 2022, and focused on understanding and mapping upstream components of the urban development system. The research team gathered and analysed data from 123 interviews, four systems mapping workshops, and two researchers embedded within local authorities to gain a comprehensive understanding of health's role in the urban development system. The systems mapping workshops involved the mapping of the complex system of urban development and the role played by health and health equity within. The participants included a diverse range of stakeholders, such as local authorities, developers, central government officials, real estate managers and investors, local communities, development consultancies, land promotion agents, and social housing organisations. A key result of Phase 1 was the identification of 50 potential intervention areas, which were then refined to seven for Phase 2. In addition to the intervention described in this paper, these interventions include: national government valuation mechanisms, city-region transport planning, law and health impact assessment, city-level spatial planning, real estate investment, and new forms of public engagement. Phase 2 started in June 2022, with a focus on designing and implementing these interventions. This paper reports on the development of the ‘Changing Mindsets’ intervention, which aims to increase the intention to act on health and health inequalities by professionals working in the private sector of urban development.

## Methods and results

### Overview of the development and optimisation process

**Fig. 1:**
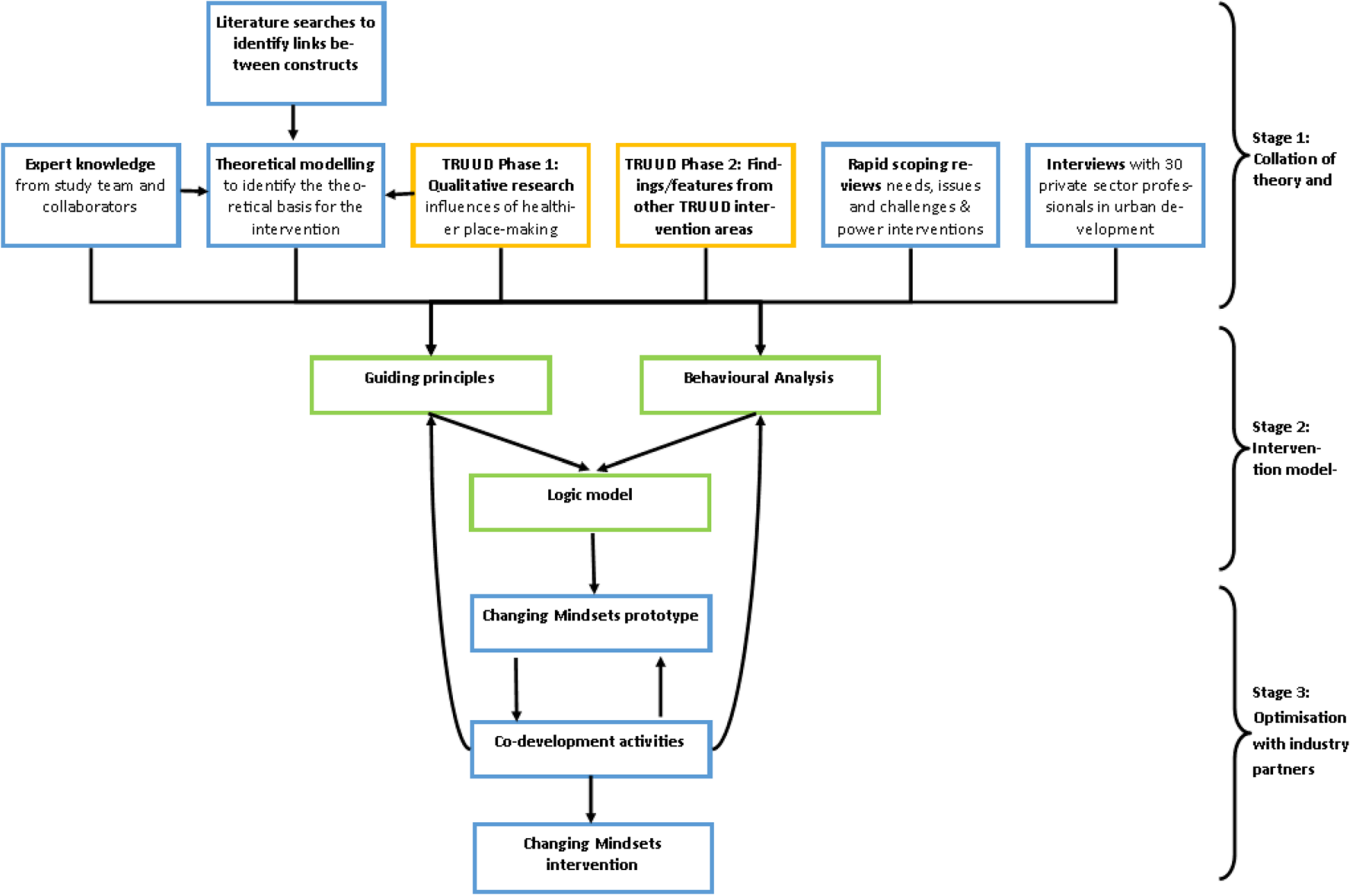
Overview of the methods used to develop the changing mindsets intervention

The Changing Mindsets intervention was developed through an iterative co-production process using the Person-Based Approach method and following Medical Research Council (MRC) guidance that interventions should draw on the latest evidence and be guided by theory [19–21]. The intervention aimed to increase the intention to act on health by professionals in the private sector of urban development with a focus on consultants and developers who were not already focussing on health as a central part of their role (hereafter referred to as the target group). Intervention development consisted of three stages: Stage 1 involved the collation of theory and evidence, which included the development of a novel theoretical framework, primary mixed methods research, and stakeholder engagement; Stage 2 was the intervention modelling phase, where the findings from Stage 1 were integrated through the guiding principles and behavioural analysis tables, which informed the logic model; and Stage 3 involved iterative intervention optimisation with members of the target population. A novel theoretical framework was necessary to bring together related but hitherto unconnected variables shaping intention to act, and thus creating a robust interdisciplinary foundation for the intervention. An overview of the development process is presented in Fig. 1.

### Intervention development and delivery team

The intervention co-design involved a collaboration between the Changing Mindsets research team, advisors from the wider TRUUD team with expertise in urban environments and health impact assessments, and two industry partners who are decision-makers within private sector urban development. The interdisciplinary Changing Mindsets research team included academics with skills from management, environmental psychology, and public health. The industry partners were recruited from a qualitative study that was conducted to explore the baseline views of the target group to support the development of the intervention and survey items. Potential industry partners were identified on the basis of holding a mid to senior position within their organisation, working with an organisation that is well known within their industry, and demonstrated interest in prioritizing health within their own professional practice. Each of these three inclusion criteria were identified from their interview content. Stage 1 and the initial intervention modelling in Stage 2 included input from the Changing mindsets team and TRUUD advisors. Stage 3 involved in depth exploration of an intervention prototype, and was followed by iterative optimisation of the intervention, and adaptation of the guiding principles, behavioural analysis tables and the logic model. The industry partners then delivered the intervention at urban development events hosting professionals from the private sector. The Changing Mindsets team attended the event to support the running of the session and to answer questions related to the content of the session.

### Ethical approval

Ethical approval for this study was received from the University of Bristol’s Research Ethics Committee on 05/01/2024 (ref: 6402).

#### Stage 1: Collation of theory and evidence

Stage 1 involved the collation of theory and evidence. This included a) the development of a theoretical framework, b) primary research exploring the evidence of needs, issues and challenges of the target group through a rapid scoping review, and 30 qualitative interviews with decision-makers in the private sector of urban development, and c) identification of potential intervention features through a literature review of existing interventions addressing systems where people did not feel powerful to effect change, in discussion with TRUUD team members from each of the six other intervention areas.

### Theoretical framework

Our theoretical framework for the intervention utilised a systems thinking method to integrate literature from the psychology of decision making and the sociology of power to better reflect the complexity of factors influencing decisions to act. There are many different understandings of the terms ‘systems thinking’, ‘systems thinking methods’ and ‘systems approaches’, which require more space to fully define. A useful introduction to this topic can be found in a 2015 Arnold and Wade (2015) paper, which defines it as: “*a set of synergistic analytic skills used to improve the capability of identifying and understanding systems, predicting their behaviors, and devising modifications to them in order to produce desired effects.”* [22].

The theoretical framework is based on insights from Phase 1 interviews and subsequent literature reviews. Phase 1 interviews indicated that inertia in the target group stemmed largely from feelings of powerlessness, a belief that colleagues and others in their industry did not care about health, and that dominant norms within the industry supported a ‘business as usual’ logic that did not take health meaningfully into account. On the basis of these findings, we conducted in-depth literature reviews to better understand how different dimensions of power (resource-based, confirm-structuration, and knowledge-based) interact with normative triggers to influence mindset shifts. These literatures include the psychology of decision-making, particularly normative messaging and group dynamics [23–26], as well as contemporary theories from the sociology of power, particularly those describing how power is created, maintained and destroyed [27, 28]. We then also incorporated theories of health and health inequalities [16] and behaviour change (i.e. the theory of planned behaviour, Ajzen 1985 [29]) to help identify the variables shaping intentions to act on health and health equity within urban development. Systems mapping techniques were used to map these variables to understand how the system of decision making functions [30, 31]. As such, the intervention aimed to use behavioural science tools (e.g. reinforcing norms related to pro-health attitudes and behaviour, shaping in-group discourse) to position health more centrally within the mindsets of professionals working in the private sector of urban development. When applying the theory in intervention development, we focussed on the higher order constructs of a) collective efficacy, comprised of empowerment, social control and social cohesion, b) power, comprised of resource-, and knowledge-based power and confirm structuring, c) proximity, comprised of emotional and cognitive proximity, and d) norms, comprised of injunctive and descriptive norms utilised within the industry and health.

### Exploration of the evidence of needs, issues and challenges of the target group

Two pieces of primary research were conducted to explore the needs, issues and challenges of the target group: 1) a rapid scoping review, and 2) qualitative interviews conducted with decision-makers in the private sector of urban development.

### Scoping review

A rapid, mixed-methods scoping review was conducted to collate existing evidence of needs, issues and challenges to decision-makers in the private sector of urban planning when prioritising health and health inequalities. We followed Arskey and O'Malley's five stage framework [32]. Searches were conducted in Scopus (619 hits) Medline (Ovid) (339 hits), Directory of Open Access Journals (3 hits), Cochrane reviews (0 hits) and Google Scholar using the search terms health* AND decision* AND urban*. A search of the reference lists of included papers was also conducted to identify additional studies. Qualitative and quantitative studies were included, and thematic analysis was then conducted on the extracted data, where the key findings were organised into themes that reflected the behavioural and psychosocial issues, needs and challenges that needed to be addressed when developing the intervention [20].

23 papers were downloaded into Endnote for full-text review and 7 papers were ultimately included. Three were produced by the TRUUD research team [17, 33, 34]. Brief details about the studies and the main barriers and facilitators to incorporating health into urban development identified in the paper are summarised in TA 1 and the nine key themes that emerged from the literature are summarised in TA 2, both available in the Supplementary material. These were challenges resulting from: the absence of a definition of a broad definition of health including the wider determinants of health***,*** location of power, absence of shared norms, language and values, competing priorities (short-term profit over long-term health), and risks of claiming healthy placemaking, in addition to the need for: advocates/champions, accessible, convincing evidence, and examples of actionable interventions. These findings were integrated into the guiding principles and behavioural analysis tables (TA 3 and TA 4 in the Supplementary materials).

### Qualitative exploration of baseline views within the land and development industries

Qualitative interviews were conducted by videocall with 30 industry professionals to explore potential influences of decision making identified in the theoretical framework that we aimed to target in the intervention, but for which no appropriate validated survey items existed. These included norms, knowledge-based power and aspects of social control. The interviews were also used to explore early ideas for the Changing Mindset intervention. Participants were recruited through existing contacts within TRUUD, contacts developed as part of an earlier phase of the TRUUD research, and snowball sampling. Interviews were semi-structured and led by a flexible topic guide that had been developed based on extensive literature searches around power as knowledge, social control and norms, as well as consultation with individuals with academic expertise in these areas. The focus on knowledge-based power and social control was based on the fact that insufficient measures exist on these topics suitable for the intervention survey. The topic guide was revised following piloting with five members of the broader TRUUD team to check for question clarity, to ensure questions were generating expected information, and to help improve consistency between multiple interviewers. The interviews lasted between 37 min and 1 h 41 min.

The interviews were conducted and coded by two team members. Thematic analysis was an iterative, ongoing, and abductive process [35]. Deductive codes were established before the analysis began, and inductive codes were developed through a process of systematic analysis and researcher interpretation [36]. The coding process involved three stages: 1) independent coding of a subset of transcripts by two researchers; 2) discussions between the researchers to refine emerging codes and develop the code hierarchy, ensuring a shared understanding and consistent coding; and 3) double-coding by a third, senior team member with extensive experience in qualitative analysis. The findings informed the development of the guiding principles, behavioural analysis tables, prototype of the intervention (webpages and presentation) and the logic model, which are reported in depth below.

### Identification of potential intervention features

Potential intervention features were identified through: 1) A literature review of existing interventions that sought to address systems where people did not feel powerful to effect change, and 2) discussion with interdisciplinary TRUUD team members from each of the six other intervention areas.

### Literature review of power interventions

A literature review was conducted to identify any intervention features that had been effective in supporting individuals to generate their own power in organisations. Searches were conducted using Google Scholar, and identification of potentially relevant interventions from reference list searches. The review explicitly sought interventions from feminist theories of power within the management literature to ensure that, in addition to the traditional ‘resource-based’ views of power that dominate organisational empowerment views, ‘relational’ views of power were clearly incorporated [37]. Search terms included ‘power interventions’, ‘power dynamics interventions’, ‘shifting power in organisations’, and ‘shifting power relations’. No relevant interventions were identified from the general Google Scholar search. The power intervention papers in management journals largely focussed on empowerment of employees in which power was ‘given’ from those at the top of the power hierarchy to their subordinates, often ‘empowering’ them through giving them more responsibility [38, 39]. Other papers focussed on ‘empowerment’ through the destruction of power hierarchies in organisations as the best method of creating power for employees [40, 41]. These intervention papers do not align with our view of empowerment in which power cannot be given from the powerful to the powerless and the destruction of power hierarchies was not a feasible task. Instead, our intervention takes the view that power needs to be created and maintained by the individual, with assistance from alterations in social and organisational structures, rather than viewing power as something that needs to be given from those with to those without. We ensured that we worked to make space for the creation of empowerment by those in the room rather than the empowerment of individuals through ‘handing over’ power from the top-down. This was done by: 1) delivering the intervention in a group setting and by industry partners to show that acting on health and health equity is an important and valuable topic in the peer group; and 2), providing opportunities for group discussion and identity and network creation through sharing of ideas and real world examples of where action on health and health equity were already taking place with peers. In so doing, this helped to reduce psychological distance, fostered a sense of in-group belonging and a group mission.

### Identification of intervention features from discussion with TRUUD intervention teams

The Changing Mindsets team met with each of the teams from the other intervention areas of the TRUUD programme to discuss how the interventions could support one another and to identify any elements that could be incorporated into the Changing Mindsets intervention. These meetings occurred on a one-to-one basis and also at full TRUUD programme consortium meetings through formal intervention coordination and integration processes. Suggestions were added to the guiding principles and discussed with the team.

#### Stage 2: Intervention modelling

Evidence and theory, stakeholder input, and expertise from the interdisciplinary team from Stage 1 were integrated in the intervention modelling phase, through the guiding principles and behavioural analysis tables, which informed the Logic model.

### Guiding principles

The ‘Guiding principles’ (from the Person-Based Approach) summarise the key intervention design needs and objectives and the intervention design features required to address these [3, 4]. In addition to these, we mapped the theoretical constructs identified in our novel framework, and the proposed mechanisms (e.g. through which intervention feature) in which they would trigger changes in intention to act on health. For example, the concept of *collective efficacy*—a key theoretical construct identified in our framework—informed the design of the intervention’s group discussions. These discussions were structured to reinforce a shared sense of purpose among urban development professionals, reducing perceived barriers to action and fostering social norms supportive of health-conscious decision-making. The guiding principles were iteratively developed using rounds of feedback with the Changing Mindsets research team. Evidence, objectives and intervention design ideas and the way they mapped to the theory were discussed and refined. The Guiding principles are presented in TA 3 of the Supplementary material, which includes the intervention design objectives and intervention features that were identified based on the evidence and theory identified in Stage 1.

The final version of the guiding principles had 10 intervention objectives:

1. Support the recognition of the power that the target group already have, and support them to foster their collective power to increase the priority of health in urban development
2. Support the development of shared norms around health and the urban environment
3. Provide evidence of the link between health and health inequalities and the urban environment, and impress the urgency of improving the urban environment in a way that connects at cognitive and emotional levels
4. Reinforce positive self-identity associated with being altruistic and doing the ‘right’ thing
5. Address the need for financial viability in the incorporation of health into urban development and provide examples of how incorporating health could increase financial viability/highlight mechanisms that could change the viability equation for developers
6. Reduce the perceived risk of claiming healthy placemaking
7. Highlight potential legal risks of not considering health in urban planning
8. Provide examples or how other urban development organisations are integrating and prioritising health and health inequalities
9. Ensure the industry insider finds the intervention easy to deliver and has buy-in to the content
10. Ensure that the potential attendees of the intervention session can see the value of the session to ensure they will want to attend

The intervention was comprised of two elements: 1) An intervention session consisting of a presentation, with group discussion, presented by one of the two industry partners. Each session was delivered by one of our industry partners who works in the private sector of urban development and discussions were moderated in part by a member of the intervention team (depending on the event); and 2) A website signposting to tools and resources, networks and support for prioritising and integrating health into urban development, and examples of how other organisations have done so. This form of intervention was chosen because a) presentations and websites are familiar and regularly used by the target group, b) the presentation with discussion provides opportunities for interaction between target group members to underscore intervention features, c) presentation content was coproduced and delivered by a target group insider and thus more likely to attract and keep attention of the target group, and d) the website reinforces the messages of the intervention, provided more in-depth information and is available as convenient for target group members. The intervention features to address these are provided in the logic model section below.

### Behavioural analysis tables

Behavioural analysis was conducted to identify behaviours to be targeted by the Changing Mindsets intervention, along with the barriers and facilitators to changing these behaviours and how this mapped to the theoretical constructs from the theoretical framework. Behavioural analysis tables were constructed using the targeted key behaviours and outcomes, with mediators of these behaviours identified from the Stage 1 evidence and theory, stakeholder input, and expertise from the transdisciplinary team. Behavioural analysis was then conducted by coding the intervention behaviours and mediators using the Behaviour Change Wheel (BCW) [42] and the Theoretical Domains Framework [43]. The Theoretical Domains Framework provides a theoretical basis for the interpretation of barriers and facilitators to the implementation of interventions in practice [43]. The COM-B model was used to code the influences and mediators of each target behaviour and intervention element onto the BCW [44]. The COM-B model proposes there are three factors capable of changing behaviour (B), which are capability (C), opportunity (O) and motivation (M) [45]. The Behaviour Change Techniques most likely to have an effect on the key target behaviours were identified from the 93-item Behaviour Change Technique taxonomy v1 [46]. The targeted key behaviours and outcomes were also mapped onto the theoretical constructs from the theoretical framework. Behaviour Change Techniques were discussed with the wider TRUUD team and industry partners to identify those that were most practical for the target group. A logic model was used to map out the relationship between intervention elements and outcomes via the expected mechanisms of change using MRC process evaluation guidance. The behavioural analysis is presented in TA 4 of the Supplementary material and provides an in-depth understanding of the behaviours targeted by the Changing Mindsets intervention and the anticipated mechanisms of change. The intervention targeted five behaviours:

- Industry partner engaging in the intervention co-design and delivery
- Target group engaging with the intervention session
- Fostering discussion and problem-solving between policy makers and the target group
- Target groups engaging with the intervention follow-up materials and information
- Engagement with research data collection

**Fig. 2:**
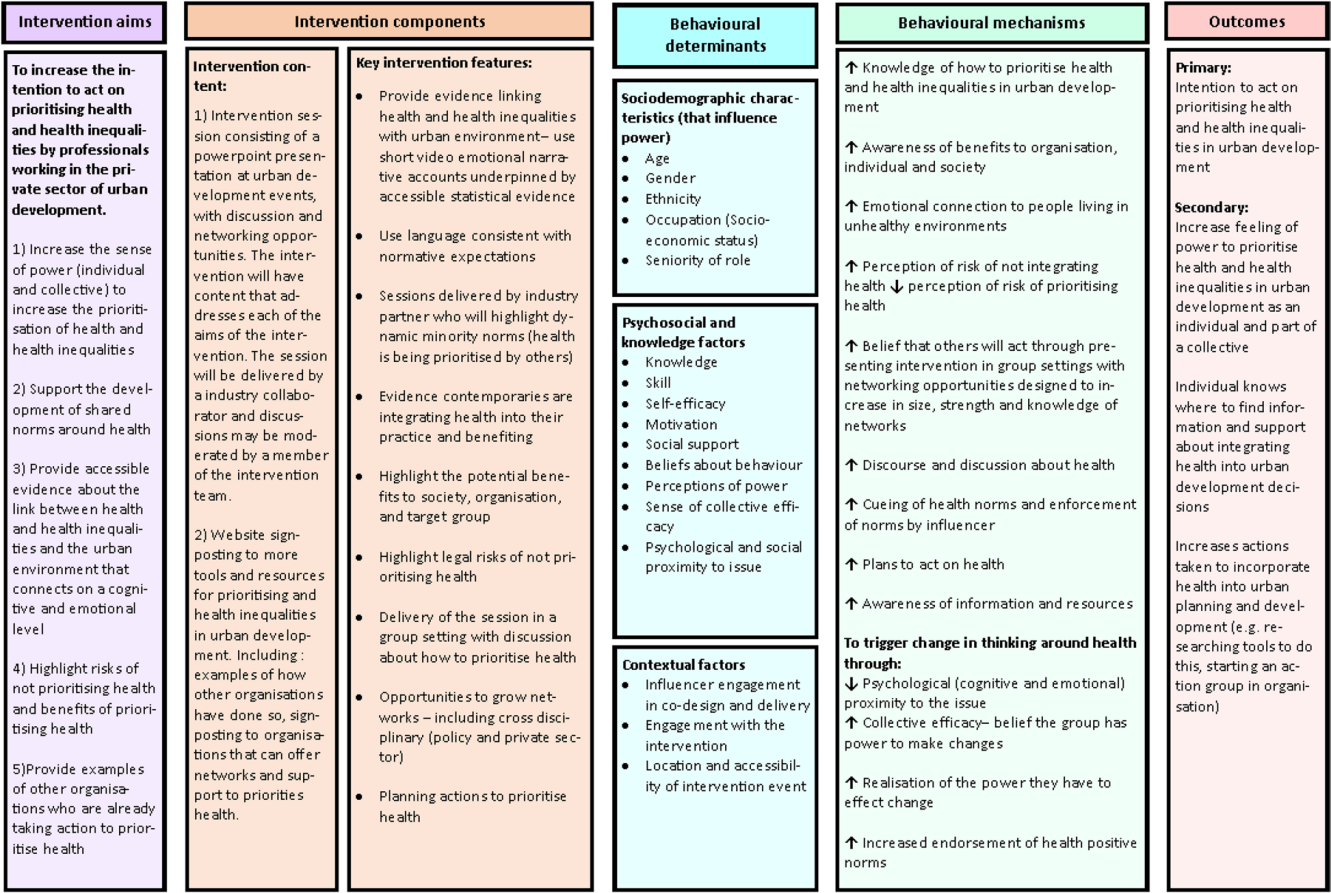
Simplified logic model for the changing mindsets intervention

These behaviours were broken down into 12 sub-behaviours that were necessary to enable them. These behaviours were mapped to 19 Behaviour Change Techniques from the behaviour change taxonomy; 12 behavioural domains from the Theoretical Domains Framework, and seven intervention types from the behaviour change wheel. The behavioural analysis informed the development of the logic model in Fig. 2.

### Logic model

The findings from the guiding principles and behavioural analysis tables were used to develop the logic model. A simplified version of the Changing Mindsets logic model is provided in Fig. 2, which provides an overview of the intervention aims, intervention components (content and features), behavioural determinants, behavioural mechanisms (including the influence of the theoretical constructs) and outcomes. The full version of the logic model is provided in Fig. 3 in the Supplementary material which also includes details of how the intervention processes map onto the Theoretical Domains Framework, behaviour change wheel and Behaviour Change Techniques from the Behaviour Change Technique taxonomy.

#### Stage 3: Iterative intervention optimisation

The aim of Stage 3 was the iterative optimisation of the intervention through co-design to ensure the design of the Changing Mindsets intervention was acceptable, feasible, interesting, persuasive and easy to engage with for the target group and the industry partners delivering it [20]. Throughout the optimisation process all feedback was added to the ‘table of changes’ and modifications were made if they were likely to have an impact on behaviour change or a precursor to behaviour change (e.g. acceptability, feasibility, motivation, engagement). Proposed changes were coded to indicate importance using the framework proposed by Yardley et al. (TA 5 in the Supplementary material) [20]. Modifications were then prioritised based on the MoSCoW (Must have, Should have, Could have, Would like) criteria [47, 48]. Proposed modifications were discussed regularly with subject experts in the wider TRUUD team to help identify appropriate modifications in response to problems identified by industry partners, or when conflicting changes were suggested. The behavioural analysis tables were regularly checked to ensure all key elements were retained in the intervention following the amendments and were updated along with the guiding principles and logic model.

### Optimisation prior to the events

First intervention prototypes were developed for the PowerPoint presentation and the website by the intervention team based on the intervention modelling stage. The prototypes went through several rounds of feedback within the Changing Mindsets team to ensure that the behaviour change features were reflected in the content before being shared with the industry partner and their team. The industry partners were also sent a link to the prototype of the website with questions to guide feedback. ‘Think aloud’ sessions were undertaken with each industry partner individually to explore initial impression of the PowerPoint presentation. The industry partner was taken through each slide and asked to speak aloud their initial impressions and were given time to ask any questions afterwards. Sessions were guided by a topic guide that prompted participants to reflect on the pros and cons of each element of the intervention. The sessions lasted up to two hours via Teams and were video recorded and transcribed using the Teams automatic captioning service. The comments were transferred verbatim into the table of changes and the team decided on modifications based on MoSCoW. Notes were made in the think alouds about what content should be moved to the script from the slides.

The new version of the PowerPoint presentation was then explored in an hour long meeting with both industry partners, where any contradictory feedback was discussed and clarification on points was requested. The Changing Mindsets research team sought consensus in this meeting for items to include or exclude, and any wording that had been queried. The amended slide deck and accompanying script were returned to the industry partner for comment. Feedback was added to the table of changes and used to revise the slide deck, the guiding principles, behavioural analysis table and logic model.

Both industry partners had a practice and feedback session ahead of the first in-person event. Both used the session to talk through the types of content they planned to include, rather than formally presenting. There was a discussion between the intervention team and each industry partner about how to strengthen some of the messaging so that it would emphasise the behavioural targets of the intervention. Following the session, the master script and presentation were amended ahead of the first event (July 2024). Each industry partner independently turned the script into bullet points with the key talking points ahead of delivery. Each industry partner was offered a technical run through for the first online event, where aspects of the intervention delivery were explored, such as the use of digital voting software, and moving participants into breakout groups for discussion.

### Delivery and optimisation during the events

It was not possible to deliver the intervention in exactly the same way for each session, because the events had different structures (e.g. online, large conference, small meeting) and limitations (e.g. time, audiovisual equipment). Therefore, the focus was on fidelity of function, rather than form, where it was intended that the same delivery goal would be achieved each time regardless of the form of the delivery [49, 50]. The core behaviour change components and source material remained static for the different events. Feedback on the intervention from the post-event survey and field notes were reviewed following each event and any issues were recorded in the table of changes and prioritised by the team for modification. Changes were made between the industry’s partner’s first and second event, where they were deemed to be important for improving the experience for the attendees and strengthening the messaging. A follow-up meeting was arranged with each industry partner following their first event to discuss changes made to the slides, and any changes that needed to be made to the script, to improve clarity for the attendees or to bring greater focus to behavioural elements.

### Long term plans for the intervention and theoretical framework

We plan for our intervention to become available for free online following our intervention evaluation period. We will be making the recording of webinars and the slide deck available. We also plan to explore the application of the theoretical framework to increasing intention to act on health in the urban environment by policy makers. We will follow the same methods outlined in this paper to adapt the intervention for this new target group.

## Discussion

### Findings

This paper describes the development and optimisation of the Changing Mindsets intervention, using evidence, theory, and the Person-based Approach. The intervention was co-designed with two industry partners who are decision-makers within private sector urban development. Intervention development consisted of three stages. Stage 1 involved the collation of theory and evidence. A novel theoretical framework was developed by this research group which utilised a systems thinking method to integrate literature from the psychology of decision making and the sociology of power to reflect the complexity of factors influencing decisions to act. This helped to identify the factors shaping intentions to act on health and health equity within urban development, which were used to trigger shifts in mindset in the intervention. A literature review confirmed there were no similar interventions aiming to support the recognition of the power that the target group already have, or to support them to recognise their collective power to increase the priority of health in urban planning and development. A qualitative study was conducted with 30 industry insiders to explore existing norms, knowledge-based power and aspects of social control to contribute to the development of the Changing Mindsets intervention. Stage 2 was the intervention modelling phase where the theory and evidence were integrated through the guiding principles and behavioural analysis tables, which informed the logic model. The intervention was then optimised in Stage 3 through think aloud activities and rounds of videocall and email feedback with the industry partners, and through feedback from the intervention events, resulting in the iterative adaptation of the Changing Mindsets intervention.

### Strengths and limitations

We used robust, established methods to develop the intervention underpinned by behaviour change theory, guided by the Person-Based Approach [20, 21]. Our theoretical framework for the intervention utilised a systems thinking method to integrate literature from the psychology of decision making and the sociology of power to better reflect the complexity of factors influencing decisions to act [22]. A wide range of mixed-methods evidence was drawn on from the TRUUD programme and the wider literature, in addition to primary research and stakeholder consultation. Professionals with a background in urban development were included throughout the development and optimisation of the intervention.

A limitation of this study is that while a broad range of stakeholders contributed to identifying and prioritising the intervention—through systems mapping of data from 123 interviews and four systems workshops—the detailed intervention co-design process was primarily informed by two industry partners. This approach ensured that the intervention was feasible, relevant, and engaging for the private sector professionals who were its intended audience. Given that the intervention was explicitly designed to shift mindsets within the private sector of urban development, it was not appropriate to include stakeholders such as public health officials, community representatives, or policymakers in the detailed development phase, as they do not belong to the target group. However, we recognise that their perspectives are critical for ensuring long-term integration of health considerations into urban planning and policy and we have plans to discuss the design of the intervention and the findings with broader stakeholders. Another potential limitation of this study is selection bias, as participants who agreed to be interviewed or attended the intervention workshops may have had greater curiosity or pre-existing interest in health. To address this, we actively targeted mainstream urban development professionals, including high-volume housebuilders, rather than those already engaged in health-focused practices.

The Changing Mindsets team initially experienced challenges recruiting industry partners prior to conducting the qualitative study. Several large organisations were approached, with the collaboration talks taking a year and reaching an advanced stage before falling through. We subsequently found success in recruiting from our qualitative study, where potential partners were identified from their interest in health and the project. While our industry partners were selected due to their personal interest in health, this was not necessarily an organizational priority, ensuring relevance to the broader sector. To ensure the intervention was acceptable to a wider range of the target group, we also modified the intervention in response to feedback following intervention events. Regardless of this, the intervention may not fully capture the perspectives of those completely disengaged from health considerations. Future work could explore ways to integrate a more diverse range of stakeholders, particularly those who may hold different levels of influence in decision-making and those with different levels of understanding about health and health equity, to enhance the applicability and scalability of the intervention.

## Conclusions

We have provided insights into how a complex theoretical framework can be combined with evidence of the target group’s needs, issues and challenges using established methodology from the Person-Based Approach and behavioural science. The Changing Mindsets intervention is currently being evaluated for its effectiveness and acceptability in the target population. Subsequent to this, there are plans to adapt the intervention and explore the transferability of the theoretical framework developed for the intervention to increase intention to act on other social issues and for other populations.

## Supplementary Information

Supplementary Material 1.

## Data Availability

No datasets were generated or analysed during the current study.
