# Supplementary material for: Development and optimisation of an intervention to increase the intention to act on health and health equity within the private sector of urban development: an evidence, theory and Person-Based Approach
Source: BMC Public Health. 2025 Apr 26;25:1564. doi: 10.1186/s12889-025-22559-w (PMC12032703; doi:10.1186/s12889-025-22559-w)
Supplement: Supplementary file 1 — Supplementary Material 1. [file 12889_2025_22559_MOESM1_ESM.docx]

## Supplementary material

Table 1: Studies included in the rapid scoping review with overview of barriers and facilitators to health/health inequalities being placed more centrally in the practice of decision makers in urban planning (in the private sector)

| **Study** | **Study description** | **Evidence of barriers to behaviour- Themes in bold**  (*methods to address barriers provided in corresponding brackets*) | **Evidence of facilitators- Themes in bold**  (*ways in which these could be incorporated in corresponding brackets*) |
| --- | --- | --- | --- |
| **Bates et al. (2023)** | **Study design:** Semi-structured interviews  **Aim:** To explore the types of health evidence that diverse actors find most persuasive in a complex policy system  **Method**: 132 multi-sectoral stakeholders spanning the urban development decision-making system. Using thematic analysis. Coding involved a deductive and inductive process. | **Perception that narrative accounts are biased:** Some participants expressed distrust towards narrative evidence, suggesting that certain stakeholders may intentionally distort information. Many referred to the necessity of data, statistics, and models to substantiate qualitative accounts. (*Recommendation: Use evidence-based narrative accounts alongside quantitative data.)*  **Evidence is not presented in a accessible way**: For most senior decision-makers, lengthy narrative reports are impractical. Many respondents highlighted that overly technical data is a barrier to its effective use, noting that not all decision-makers possess advanced technical skills. *(Recommendation: Provide concise narrative summaries with quantitative data in an easily interpretable format.)*  **Convincing private sector urban planners that health is their concern**: Several respondents noted that health is often not a high priority. However, when health-related costs directly affect an organisation’s finances, they are more motivated to act. *(Recommendation: Reduce psychological proximity by using emotive narratives and offering information on the commercial costs related to health. Provide a broad definition of health, showing its relevance to the urban environment and areas where the private sector operates.)*  **Health is not a top priority**: Investors and developers prioritise decisions based on financial viability rather than public health outcomes. Directors are primarily focused on aligning their company's actions with its goals and increasing its value, rather than prioritising health improvements. *(Recommendation: Increase the cognitive proximity to health by highlighting the urgency and relevance of acting on health concerns.)* | **Using evidence-based data in a way that provokes an emotional response**: Evidence presented as narratives that are built on relatable, real-world examples are important across a complex system. Designing messaging that has a strong emotional impact was seen as particularly persuasive. Lived experiences were shown to be important in getting the attention of busy people and making them care enough to invest valuable time in seeking change. (*An emotive narrative will be used to take the decision maker through the problem, solution and its impacts by drawing on relatable emotive stories*.)  **Need to pair narratives with accessible quantitative data evidence**: Narratives supported by credible evidence were seen as effective only if presented in an accessible way. This was true for all, but especially for decision makers with limited time and varying technical expertise. (*Short narratives will be used coupled with accessible quantitative data*)  **Types of data**: Stakeholders in the urban development sector responded positively to health evidence that demonstrates (i) negative health outcomes and (ii) the associated costs. Data on negative health impacts, such as death rates or cases of asthma and mental health issues, tend to resonate emotionally with people. Private sector actors showed a preference for evidence that highlights the economic impact of unhealthy urban development, especially when a commercial benefit can be identified. (*Potentially include information about commercial advantage of incorporating health- though care must be taken as this has the potential to increase health inequalities through the ‘green premium’*) |
| **Black et al** (2021)  Overcoming Systemic Barriers Preventing Healthy Urban Development in the UK: Main Findings from Interviewing Senior Decision-Makers During a 3-Year Planetary Health Pilot | **Study design**: Qualitative- semi structured interviews  **Aim**: Investigate the main barriers and opportunities for creating healthy urban environments.  **Method**: Two rounds of semi-structured interviews were undertaken with 15 senior decision-makers from the UK’s main urban development delivery agencies, both public and private. | **Short-termism (short term profit over long term gains):** Short term financial horizons (i.e. shareholder expectations and six-monthly reporting; sale of public assets for short-term gain). (*Highlight the urgency of placing health and health inequalities more centrally in planning reducing cognitive proximity/highlight potential of leaving a legacy)*  **Absence of ideas about how to effect change**: (*Need to provide links to tools/frameworks and examples of good practice*)  **Scepticism of current methods** **demonstrating social value:** Social valuation methods are being used by some private sector actors primarily as marketing, and possible negotiation, tools. (*Highlight examples of best practice that is not tokenistic*)  **Land banking:** Keeping hold of land so it raises in value e.g. waiting for planning permission/infrastructure to be improved (*cannot be addressed in our intervention*) | **Knowledge of the issue**: The majority of urban health challenges are well known to decision-makers, including air pollution, excessive car use, obesogenic food environments, mental health, and the need for access to nature. (*Provide evidence linking health outcomes to wider determinants rather than more obvious/known connections*)  **Tools**: Interested in tools to support prioritising issues, understanding orders of magnitude and communicating value (*Highlight tools that can be used*)  **Need for shared language and values**: (*Provide opportunities for different stakeholders to meet/join networks and discuss how to integrate health into their practice. Highlight best practice integrating health into the masterplan as an opportunity to share goals and language around health*) |
| **Carmicheal et al** (2012)  Integration of health into urban spatial planning through impact assessment: Identifying governance and policy barriers and facilitators | **Study design**: Literature review  **Aim**: Examining the barriers and facilitators in integrating health in spatial planning at the local, mainly urban, level, through appraisals.  While the focus is on the integration of health into urban spatial planning through impact assessment- there are general findings about integrating health into planning that can support our intervention planning. | **Overly narrow definition of health:** Is a major barrier to the better integration of health into the spatial planning system. Appraisals of planning proposals tended to focus on physical and environmental health concerns, such as air, water and noise issues, rather than on the broader social and cultural determinants of health. (*Provide the Wider determinants of health definition*)  **Lack of partnership working:** A lack of cross-sector understanding, including limited knowledge of planning by health professionals and vice versa, creates discomfort among partners and undermines trust. This, in turn, hampers the potential for future collaboration. Several authors highlight the constraints posed by limited time and human resources available to invest in building effective partnerships, which also affects the ability to conduct community engagement and participatory stakeholder workshops. (*Provide opportunities to network with people from different sectors and signpost to existing networks*)  **Different language and cultures:** Organisations who need to work together to integrate health into planning often have different cultures, and use different languages and terminologies. Causing issues with interpretation and contextualisation of key terms such as health. (*Provide definitions that can be used and shared and opportunity for discussion to establish a shared language and purpose*) | **Provide broad definition of health**: Using a broad definition of health should drive partnerships between health and planning professionals, better quality and range of evidence considered and refocus the problem definition towards the consideration of positive health impacts of plans/projects. (*Provide broad definition of health*)  **Use of data:** To provide the evidence of the health problems in localities, and to explore causal pathways connecting health outcomes to wider determinants. (*Provide evidence linking health outcomes to wider determinants*)  **Need for shared language and values:** Creating improved participatory models and inclusive partnerships, grounded in shared language, can facilitate the integration of diverse knowledge, expertise, and life experiences. This approach can also support the development of broader problem definitions that take into account the health impacts of planning decisions. (*Provide opportunities to network and join networks)*  **Provide training:** multi-agency training courses should be developed, to improve knowledge and understanding of health and to bring those partners together for shared learning and development of relationships (*Providing training workshops, with opportunities for cross-sector networking*)  **Partnership working:** Several studies identify the need for close partnership working from an early stage, including the development of a shared vision among partners through coalition and consensus building, formalised arrangements for partnership working, and explicit roles and responsibilities (*Highlight as example of good practice*) |
| **Chang et al** (2018)  TCPA  Securing constructive collaboration and consensus for planning healthy developments A report from the Developers and Wellbeing project | **Study design**: Workshops and interviews  **Aim**: This project explored how we can encourage a consensus between the public and private sectors and wider stakeholders about the need to build and sustain high-quality, healthy places.  **Methods**: Ten workshops with local authority planning and public health teams, housing developers and a wide network of stakeholders. Developers were interviewed to find out what they thought about their role in creating healthy places. | **Definition of health**: Generally developers are more comfortable talking about “wellbeing” rather than “health”. “Health”, to many people, means doctors and dentists, rather than the wider determinants of health. (*Provide definition of Wider determinants of health*)  **Believe they are already addressing health needs:** Developers involved in the project, either directly or indirectly, asserted that they were already acting on health, while some councils working with them strongly disagreed. It is possible that developers addressed the provision of healthcare facilities but were unaware that the councils also expected them to address broader public health concerns and the "wider determinants" of health through the design of the development. (*Provide a the Wider determinant of health definition and evidence to link health with these broader determinants*)  **Expectation that healthy environments cost more and reduce margins**: Developers were already familiar with and acknowledged the principles and guidelines for healthy development. The challenge lies in convincing them that "high quality" doesn't have to equate to "high specification" or "hard infrastructure," which often result in increased construction costs and, ultimately, higher house prices. (*Highlight alternative viability calculations)* | ***Supporting the argument for integrating health into urban planning:***  **Leaving a legacy**: (*Provide examples of companies that are looking to leave a legacy*)  **Premium associated with healthy environments**: developers are aware that they can add value to their developments by including “healthy” elements in them.(*To avoid contribution to the ‘green premium’-Provide information about alternate viability models- other benefits to the organisation of prioritising health e.g. reputation)*  ***Examples of how to integrate health into urban planning:***   - **Draw attention to existing sources of health evidence** - **Early involvement of stakeholders** - **Industry initiated standards demonstrating health:** - **Tools and frameworks**: - **Consensus on what a healthy development is and the key mechanisms for delivering it** (*Providing a definition of health and best practice examples*) |
| **Le Gouais et al.** (2023)  Understanding how to create healthier places: A qualitative study exploring the complex system of urban development decision-making | **Study design**: Qualitative semi-structured interviews  **Aim**: Explores how to influence healthier place-making with urban development decision makers  **Methods**: 132 multi-sectoral stakeholders spanning the urban development decision-making system. Using thematic analysis. Coding involved a deductive and inductive process. | **Where the responsibility lies for health**: Many stakeholders did not seem to view health as their responsibility. (*Highlight where they are able to make an impact*)  **Lack of perceived power:**  Private sector actors appeared motivated to improve people's health but felt they had limited power to integrate health into urban planning. (*Support the feeling of power through collective efficacy*)  **Old boys’ network:** Accusations of an ‘old boys’ network’ in some private sector organisations (*support the development of networks and collective efficacy to create the groundswell for change*)  **Knowledge**: Absence of a definition of health - shared language (*Provide definition of Wider determinants of health)*  **Competing priorities vs long termism (profit vs health)**: Private sector priorities tended to focus on maximising profits, which could conflict with healthy urban development outcomes. However, it appeared that some elements of the market (e.g. long-term and institutional investors) were increasingly needing to consider sustainability and wellbeing issues. (*Use mechanisms to increase proximity to the issue of health to increase the salience and urgency in their minds*)  **Issues with existing tools to demonstrate healthy spaces**: Some voluntary certifications were said to increase rental yields by attracting occupiers (e.g. BREEAM or WELL certification). However, it was also suggested that “you can cherry-pick which [standard] you want to use” and therefore it was easy to game. (*Provide best practice examples of how standards have been applied*) | **Broad definition of health**: (*need for a shared definition of health and definition of a ‘healthy’ urban development*).  **Data associating health with the urban environment:** A quantitative measure of health, similar to carbon metrics was described as potentially “hugely helpful” and monetising health benefits was seen as useful by some. (*Provide training including best practice examples of healthy urban development and signpost to HAUS model*))  **How to present the evidence**: Interviewees discussed “choosing the right language and the right argument for who you're trying to talk to”, which can involve both qualitative case studies and quantitative data. (*Present data as qualitative and quantitative, co-develop intervention with target group to ensure language is not off putting*)  **Power**: Understanding influences across the complex system may challenge beliefs of some investors and developers who thought they had a limited role in shaping health outcomes. (*Draw on collective efficacy to support the feeling of power to change*)  **Cost of healthier spaces (implications for inequalities):** Increased demand for healthier places could result in a ‘health premium’. (*Careful consideration needs to be made about the inclusion of information associating incorporation of health into planning with increased sales prices - may need to directly address the implications of this for health inequalities*).  **Long-termism (creating a legacy):** Some developers were “in it for the long term” which may increase consideration of health and wellbeing issues. (Highlight the legacy they are leaving)  **Champions/advocates**: ‘Relational capital’ was discussed as a way to initiate projects – people “that have really cared and have been really willing to drive it and eventually you bring everyone else” (*Develop buy in to prioritising health in urban planning by developing a ground swell of advocates through the intervention*) |
| **Pineo et al.** (2022)  Built environment stakeholders’ experiences of implementing healthy urban development: an exploratory study | **Study design:** Semi-structured interviews  **Aim**: To explore how the principles of healthy urbanism are put into practice through investigating built environment professionals’ experiences of implementing health in new urban developments.  **Methods**: Interviews with 31 built environment and public health professionals involved in such projects in Australia, China, England, the Netherlands, Sweden and the United States | **Gaps in knowledge of how to incorporate health** (*Highlight how others in the industry have integrated health into practice*)  **Outcome vs output driven** (short vs long-termism): Organisations more willing to take a healthy design approach were described as *outcome-driven-* with a long term approach to development. Those who are not described as *output-driven*. Both outcome- and output-driven developers were focused on finances. There was a perception that outcome-driven developers *want* homes that support health, sustainability, etc and want to get a good price for them. However, their outlook towards these goals means that health-promoting design is more likely to be integrated, within financial limits. (*Acknowledge the challenges that some of the target group target will have in shifting the focus of an output-driven organisation*)  **Risks to claiming healthy placemaking (e.g. lack of control, unknown costs, lack of expertise) *(****highlight that we are aware of the risks and how these can be mitigated****):***  **Communicate value and cost:** the current system does not allow developers from accounting for the wider economic costs and benefits of prioritising health in their financial viability calculations. (*Provide alternative cost-benefit analysis; HAUS*)  **Power**: There is an unresolved question as to whether professionals can ‘convince private sector developers’, with arguments about wider value. Pointing to inherent power differentials between developers and other . In addition to pragmatically evaluate the mechanisms that could change the viability equation for developers. (*Address power through collective efficacy & and introduce HAUS model to support alternate viability calculation*) | **Need for evidence linking design with health improvements**: Developers noted there was not always evidence available to demonstrate the added value of healthy developments. Making it difficult to build a *‘business case’*. *(Signpost to tools and information that demonstrates the link between health and urban planning)*  **Shift from short-term cost to long-term gain:** There was an indication of a recent shift in developers’ conceptualisations of economic value, from short-term costs to long-term gains, thereby affecting how design teams can make a business case. (*Highlight benefit of healthy places for legacy of the company*)  **Advocacy**: Participants spoke of discussing, persuading, negotiating, and influencing to bring forward specific healthy design measures. An American planner said *‘ … it’s all* (*Industry partner delivered intervention, and intervention that draws on collective efficacy*)  **Network building:** mechanisms to change power dynamics through networks and partnerships. Professional bodies and informal networks were described as key factors for sharing good practice and building capacity in the sector. (*Support network building, development of a shared language, collective efficacy*)  **Reflective and evaluative practice:** Participants use knowledge from experience and *‘intuition’*, in addition to evidence and research. Healthy development is an iterative process in which participants attempt to solve a problem, create a solution, critique that solution, and then take forward that knowledge in future practice. This responds to the ‘knowledge gap’ in integrating health into development, through reflective practice. Collaborative and shared knowledge was also used to achieve reflective practice. Bringing together professionals with diverse types of knowledge can deal with the complexity of urban health. (*Support reflective practice through discussion in the in-person event, through supporting network building/joining existing networks, and providing the example of reflective practice*) |
| **Riley and de Nazelle** (2018)  Barriers and Enablers of Integrating Health Evidence into Transport and Urban Planning and Decision Making | **Study design**: Literature review:  **Aim**: Highlights some of the barriers to integrating health into urban and transport planning and policy with the aim of suggesting opportunities through which these barriers may be overcome. | **Narrow understanding of health:** (*Provide definition of Wider determinants of health - and examples of how urban planners can influence wider determinants of health*)  **Translating evidence for urban planners**: Evidence often fails to effectively communicate the complex relationship between the urban environment and health - complexity so that decision makers are left with only a partial understanding. (*Provide qualitative and quantitative evidence together and an example using the HAUS model*)  **Lack of inter-sector collaboration**: (*Supporting networking*) | **Use of stories and case studies**:  Policymakers generally perceive stories and case studies as more compelling evidence than rigorous studies or literature syntheses. (*Provide case study with quantitative evidence*)  **Providing potential solutions**: Researchers could also work to better communicate their findings and suggest interventions that are actionable. (*Provide examples of how other organisations have prioritised health in their work*)  **Tools to support:** Evidence that provides a costing and an analysis of links between urban design features and health. (*Signpost to a costing tool - HAUS*)  **Intersectoral collaboration**: Could be encouraged through formal and informal structures. (*Support the development and strengthening of networks*) |

Table 2: Themes from the scoping review related to the behavioural/psychosocial issues, needs and challenges to incorporating health into urban planning

| ***Key themes*** | **Detail of the issue, need or challenges from the literature** | **Recommendations to address the issue, need or challenge from the literature** |
| --- | --- | --- |
| ***Absence of agreed broad definition of health*** | **Lack of agreement on the definition of health:** The urban development sector has a tendency to focus on physical and environmental health concerns, such as air, water and noise issues, rather than on the broader social and cultural determinants of health (17, 49, 50).  **Where the responsibility lies for health**: the narrow definition of health leads to urban developers not viewing health as their responsibility (17, 51).  **Believe they are already addressing health**: Due to the narrow definition of health, some developers felt they had already consulted on health but had not had any input from the public health team, or they had not addressed the range of public health concerns and the “wider determinants” of health expected by the council (50). | **Provide a definition of the wider determinants of health** (49). |
| ***Position of power*** | Private sector actors appeared motivated to improve people's health, but felt they had limited power integrate health into urban planning (17). There were accusations of an ‘old boys’ network’ in some private sector organisations that shaped decision-making (17)  There is an unresolved question in Pineo et al.’s (2022) interviews as to whether professionals can ‘convince private sector developers’, with arguments about wider value. They concluded that this points to inherent power differentials between developers and other stakeholders (52). | None |
| ***Competing priorities - short term profit over long-term health*** | **Health is not as high on the agenda as financial gain**: Investors and developers make decisions based on financial viability and increasing the value of the company as opposed to improving health outcomes (17, 35, 36). Where associated health costs have a direct financial impact on an organisation, they are energised to act (35, 36, 52).  **Challenges of the ‘green premium’:** Developers are aware that they can add value to their developments by including “healthy” elements in them. However, higher prices due to ‘green premiums’ may exacerbate inequalities (17).  **Outcome vs output driven**: Organisations more willing to take a healthy design approach were described as *outcome-driven* and those who are not described as *output-driven*. Both developers were focused on finances, however the outcome-driven companies are more likely to integrate health-promoting design, within financial limits (52).  **Shift from short-term cost to long-term gain:** There was an indication of a recent shift in developers’ conceptualisations of economic value, from short-term costs to long-term gains, thereby affecting how design teams can make a business case (17, 52).  **Communicate value and cost:** There is evidence that high-quality development creates value for health, social, economic and environmental outcomes. However, market structures prevent developers from accounting for these wider economic costs and benefits in their financial viability calculations (52). | **Highlight benefit of healthy place for the organisation**- for legacy and reputation of the company and provide examples of other companies who are doing this(50)  **Propose alternate viability models:** There is a need to pragmatically evaluate the mechanisms that could change the viability equation for developers (7). |
| ***Absence of shared norms, language and values*** | **Lack of intersectoral collaboration**: Has resulted in a lack of inter-sectoral understanding and knowledge, leading to a lack of trust, which hindered future partnership working (49, 51).  **Different language and values:** Various organisations who need to work together in order to integrate health considerations into planning often have very different cultures, and use different languages and terminologies. This can cause problems in interpretation and contextualisation of key terms such as health. (36, 49, 52)*.* | **Need for formal and informal networking structures** (49, 51).  **Support the development of a shared language:** Common languages can help the integration of different forms of knowledge, expertise and life experience and can in turn help identifying broader problem definition to consider health outcomes of planning decisions (49).  **Shifting norms:** Urban planners and developers spoke of discussing, persuading, negotiating, and influencing to bring forward specific healthy design measures (52).  **Develop shared values regarding health in developments** (36). |
| ***Risks of claiming healthy placemaking*** | **Risk of claiming healthy placemaking:**   - **Lack of control**: About the measurable or perceived health impacts of development (52) - **Perceived risk of costs** associated with design team knowledge gaps, expensive materials or technical systems, certification, community participation, maintenance and more. There is also is a lack of data about whether healthy buildings can achieve a higher value for commercial developers (52). - **Challenge of applying industry standards** (52). | **Provide examples of how to de-risk healthy place-making (52):**   - **Pilot projects:** Overcome the perceived risks and typical economic constraints of new development. Can be used to explore innovative practices without necessarily promising success. - **Standards**: Were seen to de-risk healthy building processes because the responsibility can be shifted to the standard itself (i.e. for both success or lack thereof). - **Cost-related risks will continue to drop over time**: As the supply chain (and potentially the public) respond to the healthy building agenda. The challenge is to make the case to developers that “high quality” does not necessarily mean “high specification” and “hard infrastructure”, all of which often lead to higher construction costs and will ultimately lead to higher house prices. - **Explore longer term return of investment**: The economic constraints and risks associated with healthy development may be dampened for developers if they retain properties and measure their return on investment over many years. |
| ***Need for advocates/champions*** |  | **Need for advocates or champions to drive the prioritisation of health** (17, 52). |
| ***Need for accessible convincing evidence*** | **Translation of evidence**: the majority of urban health challenges are well known to decision-makers, including air pollution, excessive car use, obesogenic food environments, mental health, and the need for access to nature (36). However, there is a challenge effectively communicating the relationship between the built environment and health, which is complex. So decision makers are left with only a partial understanding (51), and often unaware of how their development can make a specific impact on improving health and wellbeing (50). Developers described needing to be convinced of the added value of healthy developments, about health improvements or financial benefits from other projects. The absence of evidence made building a *‘business case’* challenging (52)*.*  **Accessibility of evidence:** providing evidence through lengthy narrative accounts is impractical, and over-technical data has been identified as a barrier to utilisation (35).  **Convincing evidence***: S*ome urban planners and developers were distrustful of narrative evidence. Data, statistics and modelling were often referred to as necessary to underpin qualitative accounts (35). | **Need to pair emotive narratives with accessible quantitative data evidence**: Lived experiences were shown to be important in getting the attention of busy people and making them care enough to invest valuable time in seeking change (35, 51). Narratives supported by credible evidence were seen as effective only if presented in an accessible way (35).  **Signpost to evidence decision-makers were interested in:** health problems in localities (49), the causal pathways connecting health outcomes to wider determinants (17, 35, 49), and evidence showing the economic valuations of unhealthy urban development, where some kind of commercial advantage can be identified (35). |
| ***Need for examples and actionable interventions*** | **Absence of ideas for how to incorporate health into urban planning:** Urban developers were interested in tools to support prioritising issues, understanding orders of magnitude and communicating value (36). They wanted suggestions for interventions that are actionable, and evidence that provides a costing and an analysis of actionable alternatives (51). | **Provide training:** Key partners should develop multi-agency training courses, to improve knowledge and understanding of health bringing those partners together for shared learning and development of relationships (49)  **Draw attention to existing sources of health evidence** (50).  **Support reflective and evaluative practice:** Healthy development involves a cyclical process where participants work to solve a problem, develop a solution, assess that solution, and then apply the insights gained to future endeavours. Utilising collaborative and collective knowledge can also facilitate this reflective practice (52).  **Support the inclusion of health in place making frameworks** (50)***.***  **Use of industry-initiated standards demonstrating health** (50). |

Table 3: Guiding principles for the changing mindsets in urban planning and development intervention

| **Intervention objective** | **Evidence/ Theory** | **Theory applied in intervention features** | **Key design features to address the issue** |
| --- | --- | --- | --- |
| **Support the recognition of the power that the target group already have, and support them to recognise their collective power to increase the priority of health in urban planning and development** | The problem -  There are three types of power that are central to the inertia around health in urban development:   - 1. Private sector actors appeared motivated to improve people's health, but felt they had limited power to integrate health into urban planning (17). - 2. There were accusations of an ‘old boys’ network’ in some private sector organisations that shaped decision-making (17). - 3. There was uncertainty about whether private sector developers can be convinced with arguments about wider value, pointing to inherent power differentials between developers and other stakeholders (52).   Potential solutions from evidence/theory:   - For point 1 - Collective efficacy has been identified as a key leverage point (32). Bandura (1977) observed that a group’s confidence in their abilities was associated with greater success (53). In the context of this intervention, if individual decision-makers in urban planning believe the group has power to make changes through their unified efforts (overcome the challenges of improving health in urban environments), they will work to do so. - For points 2 and 3 - Frame health and health equity solutions/actions in language consistent with normative expectations of mainstream group (e.g. that health can help to make money, reduce risks etc.) (54). - For point 1: Discourse is what is thought of as 'true' within a context. When less accepted meanings gain traction, this creates power. The previous definitions, norms and values that led to certain individuals or groups being powerful shift, and power no longer resides with those who created the previous meanings, values and norms(54). - For points 2 and 3- There is evidence that people tend to listen and respond more strongly and consistently to those who we view as part of our peer groups and communities (55). This also supports the reduction of social proximity as the industry partner is taking action to address issues of a socially distant target (users of the urban environment) [6] - For point 3 – reduce the proximity between developers and other stakeholders (56). Psychological proximity consists of cognitive and emotional proximities. Reduction of perceived cognitive and psychological proximity to the issue, has been associated with greater engagement with social campaigns (56). Perceived salience, knowledge, and relevance were demonstrated to be strong predictors of psychological proximity, which in turn leads to positive campaign outcomes (56). Emotional connectedness and empathy are determinants of emotional proximity (56). There is evidence that emotional intensity reduces perceived psychological distance (57). The reduction of social proximity also supports prosocial actions (58). It has been proposed that when people learn about others who help those who are socially distant to themselves, prosocial actions will become more salient because it challenges the lay beliefs about distance and helping. (58). | - Power-Resources, knowledge, confirm structuration - Collective efficacy- Social control Empowerment, Social cohesion | Supporting individuals to identify the power they already have:   - Points 1 and 2 - Enable individuals to recognise they have power in the form of choices to incorporate aspects of health into their practice e.g. investigating/researching health for themselves, encouraging the forging of connections with those who do have expertise (e.g. supporting the development of networks), applying methods and experience of other developers to their own work e.g. reflective practice, pilot work, placing a person in the organisation in charge of prioritising health   Supporting decision makers to recognize collective power to change:   - Point 1: Deliver the intervention in group settings, where individuals feel that they can make a change as part of a collective. Encourage the sharing of ideas about the issue to reduce psychological distance. Foster a sense of in-group belonging and a group mission using group discussion, time for informal networking/links to existing networks - Point 3: Provide evidence that contemporaries are already integrating health into their practice and reaping the benefits - Point 3: During discussion session- ask ‘What are colleagues in your industry already doing?’. This supports social control within the in-group, as people discuss and are made more aware of who they think is active and is acting in the ‘common good’.   Intervention delivered by an industry partner in the field of urban development:   - Points 2 and 3: Industry partners will present the invention session to address the issue of the ‘old boys’ club’ because the messaging is delivered by someone from the ‘in group’.   Running a collaborative event with the national government intervention arm of TRUUD. The event will bring together members of our target group in the private sector of urban development and policy makers- to support the formation of cross industry collaborations, increase access to information and to change Discourse around health, which could increase the sense of power in the group. |
| **Support the development of shared norms around health and the urban environment** | - **Shifting norms:** The predominant norm in urban development is often cited as the need for viability and to prioritise making a profit (baseline interviews). Urban developers spoke of discussing, persuading, negotiating, and influencing to bring forward specific healthy design measures (52). - There is evidence that **dynamic minority norms** (information about collective change in behaviour) can result in meaningful change in personal behaviour, which can occur even when the behaviour is not currently the norm **(59)** - **Shifting of Discourse** to support the acceptance of new definitions, norms and values – thereby shifting power from individuals or groups from those who created previous meanings, values and norms (54). | - Power - knowledge, confirm structuring) - Collective efficacy - social cohesion, social control - Group norms - norms around profitability (traditional) and dynamic minority norms (e.g. norms around common good as relevant in urban development) | Support the 'defining of a new Discourse' that prioritises health in the urban environment through discussion and debate.   - The industry partner will pick up a minority norm and demonstrate that it is dynamic and moving in the direction of improved consideration of health e.g. by providing examples of best practice where other organisations are already taking steps to prioritise health. - Messages will be framed around cueing particular norms (e.g. norms around profitability (traditional) and dynamic minority norms (e.g. norms around common good as relevant in urban development)   Run events that involve individuals from different disciplines (e.g. policy makers, third sector) with the target group and provide opportunities for discussion and the shifting of Discourse. This will be in collaboration with the TRUUD programme’s policy intervention. Support the sharing of ideas and support the formation of networks and new norms, by providing the opportunity for discussion at intervention events and through networks joined following the event. |
| **Provide evidence of the link between health and health inequalities and the urban environment and impress the urgency of improving the urban environment in a way that connects at cognitive and emotional levels** | ***Barriers to change***  **Narrow definition of health:** Has led to many urban developers not viewing health as their responsibility (17), or in their remit to influence (51) (Unpublished TRUUD Phase 1 data). Some felt they were already consulting on health and addressing issues, although they had not had input from the public health team, or they had not addressed the range of public health concerns and the “wider determinants” of health expected by the council (50).  **Translation of evidence**: Evidence often fails to effectively communicate the relationship between the built environment and health that is complex, context specific and dynamic. So decision makers are often left with only a partial understanding (51). In many cases developers are unaware of how their development can make a specific impact on improving health and wellbeing (50). Developers described needing to be convinced of the added value of healthy developments, about health improvements or financial benefits from other projects. The absence of evidence made building a *‘business case’* challenging (52)*.*  ***Facilitators to change***  **Convincing evidence***:* Designing messaging that has a strong emotional impact was seen as particularly persuasive. Lived experiences were shown to be important in getting the attention of busy people and making them care enough to invest valuable time in seeking change (35, 51) However, some urban developers have reported being distrustful of narrative evidence. Data, statistics and modelling were often referred to as necessary to underpin qualitative accounts (35).  **Narrative accounts can reduce cognitive** and emotional proximity to an issue, which has been associated in greater engagement with social campaigns (56). ‘Communication strategies such as framing the campaign issue as salient and personally relevant can be used to increase a person's cognitive proximity toward the issue and consequently generate greater participation intentions. Similarly, enhancing knowledge about an issue by delivering information about the benefits of resolving the issue is another way to increase cognitive proximity. A person's increased cognitive proximity can partially lead to emotional connectedness and empathy toward people suffering from a social issue, which are critical predictors of individuals' willingness to engage in a relevant campaign (56)  **Accessible evidence:** Narratives supported by credible evidence were seen as effective only if presented in an accessible way. This was true for all, but especially for decision makers with limited time and varying technical expertise (35). | - Collective efficacy - social cohesion - Power - knowledge, resources - Psychological proximity - cognitive and emotional | Features from evidence:   - To prevent alienation of target group, acknowledge that urban planners have a good knowledge of aspects of health and are already incorporating it into their practice to some degree. - Provide information on the wider determinants of health and how they influence health and health inequalities   Inclusion of information that reduces cognitive and emotional proximity:   - Pairing of short emotional narrative accounts underpinned with accessible statistical evidence linking the wider determinants of health with urban planning and development, and highlighting the urgency and increasing salience. Using the lived experience videos produced by the Public Engagement intervention of the TRUUD programme. - Provide examples of evidence linking the wider determinants of health and their impact on health inequalities to the urban environment to support understanding of the definition. Examples should be within the target user’s realm of influence and expertise, to demonstrate where they can make an impact on health (e.g. pollution, greenspaces) or health inequalities (e.g. affordable housing in healthy environments) (personally relevant information reduces *Psychological proximity*). - We may need to have the messaging repeated to increase familiarity and to encourage the formation of concrete ideas about the issue e.g. encourage sign up to the TRUUD newsletter, and accessing the website with more detailed information - Signpost to evidence linking the urban environment with health that decision-makers were interested in: e.g. the causal pathways connecting health outcomes to wider determinants and to urban design features (17, 35, 49). - Provide evidence linking health inequalities to the urban environment through examples in the lived experience video (from the TRUUD programme’s Public Engagement team’s intervention) |
| **Reinforce positive self-identity associated with being altruistic and doing the ‘right’ thing** | **Applying ethical guidelines***:* Although group norms are powerful in shaping behaviours (60), people do not act due to normative conformity in many instances (61). Often people act because they perceive the behaviour or act to be the ‘right’ thing to do (62). These behaviours are ‘sociotropic’, and rest on judgements about whether the behaviour will promote societal welfare, rather than the individual (63), and are based on an existing value system (64). | - Psychological proximity - cognitive, emotional - Power - knowledge and resources - Social norms - profit maximization | We will highlight potential benefits to society (e.g. reducing health inequalities and improving population health) or target group (e.g. by reinforcing positive self-identity, highlighting benefits to the organisation) |
| **Address the need for financial viability in the incorporation of health into urban planning and provide examples of how incorporating health could increase financial viability/ highlight mechanisms that could change the viability equation for developers.** | - Investors and developers make decisions based on financial viability and increasing the value of the company as opposed to improving health outcomes (17, 35, 36). Where associated health costs have a direct financial impact on an organisation, they are energised to act (35, 36). - Developers are aware that they can add value to their developments by including “healthy” elements in them. However, higher prices due to ‘green premiums’ may exacerbate inequalities (17). - There was an indication of a recent shift in developers’ conceptualisations of economic value, from short-term costs to long-term gains, thereby affecting how design teams can make a business case (17, 52). - Urban developers were interested in tools to support prioritising issues, understanding orders of magnitude and communicating value (36). They wanted evidence that provides a costing and an analysis of actionable alternatives when integrating health into urban planning and development (51). | - Power - knowledge - Collective efficacy - social cohesion - Psychological proximity - cognitive | Provide examples of alternative viability:   - Although the target group were interested in the commercial advantages of building healthier environments, we will not be pursuing this because higher expected prices could exacerbate health inequalities. - Instead, we can provide alternative examples of how the incorporation of health can increase the value of the company/highlight mechanisms that could change the viability equation for developers - e.g.   - Focus on long-term gain - for example by highlighting the benefit of healthy places for legacy and reputation of the company and provide examples of other companies who are doing this.   - The focus on health will gain or strengthen the reputation of the organisation for being pioneers in the integration of health into urban planning. They are more likely to become a model of best practice for healthy land disposal and/or development. This has subsequent potential benefits of being asked by government departments to help shape policy and have more favourable outcomes in interactions with governments (such as in the planning process and in writing of legislation and supporting guidance).   Signpost to sources of health evidence and tools that can help support the cost/benefit analysis of incorporating health into urban planning and development: e.g**.** produced by Public Health England and councils, such as the Joint Strategic Needs Assessment. Provide information about the TRUUD Health Appraisal of Urban Systems (HAUS) model – a tool for the cost/benefit analysis of incorporating health into urban developments |
| **Reduce the perceived risk of claiming healthy placemaking** | Urban developers described perceived and actual risks of claiming healthy placemaking (52) (Stage 1 interviews), originating from:   - Lack of control about the measurable or perceived health impacts of development (52) - Perceived risk of costs associated with design team knowledge gaps, expensive materials or technical systems, certification, community participation, maintenance and more. There is also a lack of data about whether healthy buildings can achieve a higher value for commercial developers (52). - Challenge of applying industry standards: Healthy building standards (primarily WELL) require many verifications (e.g. air quality) when the building is occupied. This results in risks for design teams, developers, landlords and tenants, with no single party being in full control of the outcome. Standards increased transparency about building performance, thereby potentially increasing risk and responsibility for some parties. | - Psychological proximity -cognitive - Power - knowledge, resources - Social norms: incorporating health is risky | Acknowledge that change can feel risky.  Decrease the perception of risk of incorporating health into urban development by highlighting the other organisations that are already acting on health and how they are doing this - these will be examples in the presentation and website. |
| **Highlight potential legal risks of not considering health in urban planning** | Information from the law intervention from the TRUUD programme Phase 2:   - Possibility of highlighting the legal risks of not considering health in urban planning. Coroners are becoming more interested in cases of social welfare and this includes explicit linking of cause of death to issues in the urban environment - e.g. mould and pollution, rather than respiratory illness and asthma. In circumstances where a death is seen to be related to the urban environment, those responsible for the conditions (e.g. Local authorities and private companies) may be required to attend court cases - Being pulled in front of the coroner can damage reputation through being criticised and this information being reported on social media, but it is also used by lawyers as an efficient way to gather evidence for civil or criminal cases, as these parties are called to respond and provide evidence in the inquest case. - Local authorities have been called to account for – poorly undertaken refurbishment, mould, air quality. - Potential upcoming changes to policy environment creating necessary changes for industry; requirement for HIA increasing across new local plans. Requirement for developers to demonstrate HIA process is conducted by individuals with appropriate credentials. | - Power- knowledge - Psychological proximity- emotional, cognitive - Suggesting alternative Social norm- more risky to not incorporate health | **Increase perception of risk not incorporating health environments to increase proximity to the issue:**   - Highlight the legal risks of not considering health in urban planning and that legal tools are changing such that these types of cases are set to increase. E.g. coroner using urban environment as cause of death - which can have reputational issues for the company responsible - Could provide examples of newspaper headlines for land control**:** Ella Kissi-Debrah (air pollution) [Illegal levels of air pollution linked to child's death - BBC News](https://www.bbc.co.uk/news/science-environment-44612642) - Housing developer example: Awaab Ishak (mould) [Death of two-year-old from mould in flat a ‘defining moment’, says coroner \| Housing \| The Guardian](https://www.theguardian.com/uk-news/2022/nov/15/death-of-two-year-old-awaab-ishak-chronic-mould-in-flat-a-defining-moment-says-coroner) - Global example of changes in legislation related to the environment: European Court of Human Rights ‘inaction on climate change violated human rights’ |
| **Provide examples of how other urban development organisations are integrating and prioritising health and health inequalities** | **Absence of ideas about how incorporate health into urban development** (36). They wanted suggestions for interventions that are actionable (51). | - Collective efficacy - empowerment - Power - knowledge, resources | - Provide and signpost to examples of where organisations have integrated wider determinants of health into urban development. - Highlight next steps integrating health in a way that makes it sound really easy to do. Depending how much experience they have integrating health into their work - Signpost to examples of how Health Impact Assessments and community health need assessments can support the consideration of health inequalities in projects. These tools can be used by urban decision-makers for local health equity monitoring processes, helping to identify, understand, access, and measure inequities - e.g. WHO Urban Health Equity Assessment and Response Tool – Urban HEART (WHO, [2010](https://link.springer.com/chapter/10.1007/978-3-030-93875-8_1#ref-CR88); Prasad et al., [2015](https://link.springer.com/chapter/10.1007/978-3-030-93875-8_1#ref-CR60); Novoa et al., [2018](https://link.springer.com/chapter/10.1007/978-3-030-93875-8_1#ref-CR55) |
| **The industry partner must find the intervention easy to deliver and have buy in to the content** | The industry partner will be a senior decision-maker in urban development. They will be very busy and therefore must be able to see the value in the delivery of the intervention for themselves and their organisation. | - Power - knowledge, resources - Collective efficacy - Psychological proximity - Shifting social norms | - The intervention must be easy to access and use for the person delivering it. There must be minimal training. The intervention will predominantly be delivered at events the industry partner was already planning on attending.   Highlight potential benefits to the individual, organisation and attendees of the project e.g.   - Develop a co-branded tool that can be used by the industry partner/organisation beyond the project. - Industry partner and organisation: Growth of reputation and esteem through being more connected to a politically important issue (health), and through improving their own knowledge base, including learning about examples of best practice. - Become more visible to policymakers and other influential people within the field - Organisation will gain or strengthen their reputation for being pioneers in the integration of health into urban planning and could become a model of best practice. - Highlight potential benefits of improving the urban environment E.g. benefits to society, benefits to the organisation (e.g. alternative viability mechanisms) or target group (e.g. reinforces positive self- identity). The intervention will be co-designed with the industry partner to ensure they are bought into the message and the delivery and it reflects the needs, issues and challenges of the target group. - The intervention will be co-branded and can be used by the industry partner/organisation beyond the project. |
| **The potential attendees of the intervention session must be able to see the value of the session to ensure they will want to attend** | Private sector actors appeared motivated to improve people's health, but felt they had limited power to integrate health into urban planning (17). They generally prioritise financial viability and increasing the value of the company as opposed to improving health outcomes (17, 35, 36). Where associated health costs have a direct financial impact on an organisation, they are energised to act (35, 36). | - Power - Collective efficacy - Psychological proximity - Shifting social norms | - Deliver the intervention at urban development events – where the target group are already planning to attend rather than those focussed on health. - The intervention sessions will be advertised through conference event schedules. At the smaller events, attendees may be emailed ahead of the event. - For the event with the TRUUD national government team - potential attendees were selected from an existing list of senior decision-makers in urban development developed in Phase 1 of the project and invited by email. - The title of the session will be co-designed with the industry insider. The summary of the session for the programme will highlight benefits of attending, e.g. we will be giving examples of:   - Opportunities to integrate and prioritise health   - Examples of where other organisations have integrated health to urban planning and development   - Tools that can support cost/benefit analysis of integrating health into urban planning - Adverts will also highlight the prestige of the industry partner |

Table 4: : Changing Mindsets Intervention Behavioural analysis table

| **Target behaviour** | **Barriers/ *facilitator* to the target behaviour** | **Evidence for barrier/ facilitator/ intervention ingredient** | **Intervention ingredient** | **Theoretical constructs** | **Target construct (BCW)**  **COM-B model** | **Theoretical domains framework** | **Intervention types (BCW)** |  | **Behaviour Change Techniques**  **(using 93 Behaviour Change Technique taxonomy v1)** |
| --- | --- | --- | --- | --- | --- | --- | --- | --- | --- |
| **Key behaviour: industry partner engaging in the project, co-design and delivery of the intervention** | | | | | | | | | |
| Agreeing to be involved and engage in co-design of the intervention | -Time  - Competing priorities - short term profits vs long term improvements in health. Not seeing the value of the project to themselves or their organisation*.*  *-Provide access to new information from the TRUUD study e.g. HAUS model, legal risks of not integrating health*  *-Provide opportunities for them to come into contact with policy decision makers involved in TRUUD*  -*Believing in the aim of the intervention*  -*Ease of engagement* | (35, 36, 51, 52, 56).  Feedback from conversations with potential industry partners and from members of the team who work in urban development indicated senior decision-makers are very busy people with lots of competing priorities and need convincing of benefits of delivering the intervention to themselves and their organisation. | Highlight potential benefits of involvement in the project:   - Benefits to society, Improving the urban environment (reduce cognitive proximity) - Benefits to the organisation (e.g. alternative viability mechanisms, reputation for being pioneers, positively viewed by stakeholders). Develop a co-branded tool that can be used by the industry partner/organisation beyond the project. - Industry partner (e.g. reinforces positive self- identity, aligns with personal values) | Power – resource-based, knowledge-based; Proximity – cognitive | Motivation (reflective);  Opportunity (Social) | Knowledge;  Social/professional role and identity;  Beliefs about consequences;  Intentions;  Goals;  Environmental context and resources;  Social influences | Education;  Persuasion;  Incentivisation;  Enablement |  | 1.9. Commitment;  5.3. Information about social and environmental consequences;  10.8. Incentive (outcome);  13.1. Identification of self as role model |
| Delivering sessions | -Lack of knowledge about the issue  -Not having bought in to content of materials.  -Subsequently having challenges guiding discussion to sufficiently change discourse, and norms.  -*Ease of delivery of the intervention*  *-Exposure to evidence that reduces psychological proximity (cognitive & emotional) to the issue*  *-Co-design materials, which involves buy-in to the issue. Multiple viewing of the material, reduces proximity and promotes familiarity with the contents*  *-Provide opportunities to discuss the issues and to present the intervention in a practice setting with feedback*  *-Have the events correspond with events the industry partner would already attend/are beneficial for them to attend* | (50, 51, 56, 57) (35, 51, 58).  Feedback from conversations with potential industry partners about events that they are going to be attending. | -Co-design intervention materials and plans for implementation with the industry partner to support buy-in and to promote familiarity with the topic.  - Have the industry partner review and feedback on all aspects of the intervention, including the lived experience videos which aim to increase psychological proximity (cognitive & emotional) with the issue.  - Develop a script with prompts for the industry partner to guide the presentation and discussions to support the delivery of behaviour change elements and shifting of norms.  - Have members of the intervention team in the room to support the discussion  - Industry partner and intervention team reflect on the successes or challenges after the session has been run- to record any thoughts about changes that need to be made before the next intervention event. | Proximity – cognitive, emotional; Group norms | Capability; Opportunity | Knowledge;  Skills;  Beliefs about capabilities;  Beliefs about consequences;  Intentions;  Social influences;  Emotion;  Memory, attention and decision processes | Education;  Persuasion;  Training;  Enablement; |  | 1.4. Action planning;  1.9. Commitment;  2.2. Feedback on behaviour;  3.2. Social support (practical);  4.1. Instruction on how to perform the behavior;  5.2. Salience of consequences;  8.1. Behavioral practice/rehearsal  9.1. Credible source;  15.1. Verbal persuasion about capability |
| **Key behaviour: target groups engaging with the intervention session** | | | | | | | | | |
| Attending the intervention session delivered by the industry partner | *-* Not knowing about the session  - Lack of interest in integrating health into urban development  - Not understanding how the session might benefit them or their work  - Not believing that they have the power to prioritise health in their work  - Not feeling they have the time to attend  - *Highlight the benefits of attending the session and how it will address some of the known barriers to integrating health into urban development for the target group.*  *- Wanting to see the industry partner present*  *-Other peers attending the session* | (17, 35, 36, 50-52, 55) | Deliver the intervention at general urban development events rather than those focussed on health. We want to make sure we are able to target people who are not yet bought into/already integrating health and health inequalities into urban development.  The intervention sessions will be advertised through conference and TRUUD communications channels to bring attention to the intervention sessions. At the smaller events, attendees may be invite only and will be emailed ahead of the event.  Adverts for the intervention sessions will be co-designed with the industry partners and should highlight benefits of attending, e.g. we will be giving examples of:   - Risks of not doing anything on health - Benefits of taking action- to society, themselves and their organisations - Examples of where other organisations have prioritised health, the challenges they faced and how they overcame them. - Highlight tools that can support cost/benefit analysis of integrating health into urban development e.g. the TRUUD HAUS tool - Adverts should also highlight the prestige of the industry partner | Power – resource-based, knowledge-based; Collective efficacy – empowerment; Group norms | Opportunity; Motivation | Knowledge;  Beliefs about capabilities;  Optimism;  Beliefs about consequences;  Reinforcement;  Goals;  Social influences | Persuasion;  Incentivisation |  | 6.2. Social comparison  10.8. Incentive (outcome) |
| Engaging with the materials/messaging during the intervention session | -Do not believe they have power to prioritise health in urban development- in part because they believe others don’t care enough to do anything, structurally there are barriers (e.g. if the client doesn’t want it, it doesn’t happen), traditional mindsets (e.g. attributed to things like an ‘old boys’ club’) preference for what has been done before with predictable outcomes, and lack of incentives for prioritising health.  - Narrow definition of health in urban development led to many urban planners and developers not viewing health as their responsibility, or in their remit to influence.  -Decisions are made that prioritise financial viability rather than health  - Evidence linking health with urban environments is often technical and complex  -Narrative accounts that are long or not supported with quantitative data are not seen as convincing  -Believe the incorporation of health into urban development and development to be risky  -Not finding the presenter convincing  *-Provide examples of how health can increase the value of the company/ highlight mechanisms that can change the viability equation for developers*  *-Increase the psychological (cognitive and emotional) proximity*  *-Industry insider deliver the intervention session* | (17, 32, 35, 36, 49-52, 54-56, 58, 59, 65)(19, 25, 41-44, 46 | - Intervention content addresses known needs, issues and challenges for the target group prioritising health:   - Highlight lesser known causal pathways between features of the urban environment and health   - Risks of not doing anything on health   - Benefits of taking action- to society, themselves and their organisations   - Examples of where other organisations have prioritised health, the challenges they faced and how they overcame them.   - Highlight tools that can support cost/benefit analysis of integrating health into urban development e.g. the TRUUD HAUS tool - Presenting information in different formats- including lived experience/ case studies accompanied by quantitative data presented in an accessible way. - Intervention delivered by an industry insider who has credibility within the industry. - The industry insider will contextualise the information for the audience to make it more relatable for the target group - Frame health and health equity solutions/ actions in language consistent with normative expectations of target group (e.g. that health can help to make money, reduce risks etc.) - Deliver the intervention in a group setting with discussion to foster shared language, a sense of group mission, and learning from the experience of others | Collective efficacy – empowerment, social control, social cohesion; Group norms; Power – resource-based, knowledge-based, confirm-structuration; Proximity – emotional, cognitive | Capability (psychological);  Opportunity (physical and social);  Motivation (reflective and automatic) | Knowledge;  Skills;  Social/professional role and identity;  Beliefs about capabilities;  Beliefs about consequences;  Intentions;  Goals;  Memory, attention and decision processes;  Environmental context and resources;  Social influences;  Emotion | Education;  Persuasion;  Incentivisation;  Training;  Environmental restructuring (social);  Modelling |  | 3.1. Social support (unspecified);  4.1. Instruction on how to perform the behavior;  5.1. Information about health consequences;  5.2. Salience of consequences;  5.3. Information about social and environmental consequences;  5.6. Information about emotional consequences;  6.1. Demonstration of the behavior;  6.2. Social comparison;  9.1. Credible source;  10.5. Social incentive;  12.2. Restructuring the social environment;  13.4. Valued self-identify ;  13.5. Identity associated with changed  Behavior ;  15.1. Verbal persuasion about capability |
| Engaging in planning actions to take to integrate and prioritise health into their work | -Do not believe they have power to prioritise health in urban development- in part because they believe others don’t care enough to do anything, structurally there are barriers (e.g. if the client doesn’t want it, it doesn’t happen), traditional mindsets (e.g. attributed to things like an ‘old boys’ club’) preference for what has been done before with predictable outcomes, and lack of incentives for prioritising health.  - Narrow definition of health in urban development led to many urban planners and developers not viewing health as their responsibility, or in their remit to influence.  -Decisions are made that prioritise financial viability rather than health  - Evidence linking health with urban environments is often technical and complex  -Narrative accounts that are long or not supported with quantitative data are not seen as convincing  -Believe the incorporation of health into urban development and development to be risky  -There is a lack of awareness of actionable interventions  *-Provide examples of how health can increase the value of the company/ highlight mechanisms that can change the viability equation for developers*  *-Increase the psychological (cognitive and emotional) and social proximity*  **-** *Motivating actors to co-operate by invoking ethical factors such as a sense of fairness, equity, and altruism*  *-Highlight risks of not prioritising health*  *-Provide examples of how other organisations have prioritised health* | (17, 32, 35, 36, 49-52, 54-56, 58, 59, 65) | - Belief that other people in the industry don’t care about health – we show dynamic minority norm (others are beginning to act on health) - Structural barriers to change and risk of prioritising health – we show how organisations and people are overcoming these with case studies and through discussion ‘*What are colleagues already doing on health’* - Traditional mindsets (e.g. risk aversion) –demonstrate dynamic minority norm - Lack of incentives to change – provide information about risks and benefits (including to society, their organisation and themselves tapping into altruism and fairness) - Provide information about the wider determinants of health and highlight where in the system urban developers have influence   -Enable individuals to recognise they have power in the form of choices to incorporate aspects of health into their practice e.g. investigating/researching health for themselves, learning from what others are doing the sector e.g. reflective practice, pilot work, overcoming systemic issues. Connecting with others who are acting on health - or want to act on health  -Supporting decision makers to recognize collective power to change:  -Deliver the intervention in group settings, where individuals feel that they can make a change as part of a collective.  -Provide evidence that contemporaries are already integrating health into their practice and reaping the benefits (supporting collective efficacy, increasing psychological proximity, demonstrating the dynamic minority norm)  -Intervention delivered by an industry partner in the field of urban development- addressing the issue of the ‘old boys’ club’ and increasing psychological proximity as the industry partner is taking action to address issues of socially distant users of the urban environment.  -During discussion session - ask ‘what are colleagues in your industry already doing?’. This creates awareness around social control within the in-group, as people discuss and are made more aware of who they think is active and is acting in the ‘common good’. (*Collective efficacy - social control*)  -Increase proximity by pairing of short emotional narrative accounts underpinned by accessible statistical evidence.  -Frame health and health equity solutions/ actions in language consistent with normative expectations of target group (e.g. that health can help to make money, reduce risks etc.)  -Highlight the legal risks of not considering health in urban development.  -Signpost attendees to more detailed information available on the Changing Mindsets website. Highlight what organisations provide networks and support to integrate health into urban development.  -Discuss with other participants solutions to raising health up the agenda, drawing on example from what others are doing  -Support the 'defining of a new Discourse' that prioritises health in the urban environment through discussion and debate.  -The discussion also supports reflective practice- where learning from others fills gaps in knowledge in the sector about how to prioritise health.  -Making plans for actions they are going to take to prioritise health:   - At in-person events provide postcards for participants to write concrete actions they plan to take to prioritise health and people they have met that they would like to connect with after the event to keep the conversation going. It will also have the QR code for the Changing Mindsets resources website. - At the online events they will be asked to email themselves with the same information and the link to the Changing Mindsets resources website will be added to the chat as well as the QR code being included on the final slide. | Collective efficacy – empowerment, social control, social cohesion; Group norms; Power – resource-based, knowledge-based, confirm-structuration; Proximity – emotional, cognitive | Capability (psychological);  Opportunity (physical and social);  Motivation (reflective and automatic) | Knowledge;  Social/professional role and identity;  Beliefs about capabilities;  Beliefs about consequences;  Intentions;  Goals;  Memory, attention and decision processes;  Environmental context and resources;  Social influences;  Emotion | Education;  Persuasion;  Environmental restructuring (social);  Modelling |  | 1.1. Goal setting (behavior);  1.2. Problem solving;  3.1. Social support (unspecified);  6.2. Social comparison;  10.5. Social incentive;  12.2. Restructuring the social environment;  13.4. Valued self-identify ;  13.5. Identity associated with changed  Behavior ;  15.1. Verbal persuasion about capability |
| Engaging in discussion during intervention sessions | - Not feeling confident to engage in discussion in groups - Not feeling that they can contribute to discussion - Not wanting to join because they don’t see it as useful - *Create an inclusive environment* - *Use language that is accessible (not jargon heavy and is well explained) and represents the group norms* | (52, 54, 56, 66) (52). | - Foster a sense of in-group belonging and a group mission and encourage the sharing of ideas about the issue to reduce psychological distance. This can be done by using breakout group discussions/time for informal networking/providing links to organisations running networks.  Create an inclusive environment for discussion:   - Allow participants to introduce themselves in small group settings - Provide multiple opportunities to engage in group discussion - Provide opportunities for participants to share ideas that have emerged from group discussion - Provide different ways for individuals to have their questions answered e.g. Q & A at the end/signposting to TRUUD contacts/opportunities to speak to the speaker and TRUUD team - Frame information and questions using language that represents group norms - Respectful moderation of discussion   Feedback on group discussion:   - In smaller groups: participant comments summarised in a feedback session through members of the group reporting back on what they have discussed - In larger groups or online events, feedback added to Mentimeter digital software - each question will have a QR code which will take them to the Mentimeter page for the question. Members of the group are asked to add their responses to Mentimeter. The industry partner will then pick out themes for discussion during the feedback session - Provide positive reinforcement for each contribution | Power – knowledge-based, confirm-structuration; Collective efficacy – empowerment, social cohesion; Proximity – emotional, cognitive; Group norms | Capability (giving them tools/language to take part in discussion);  Opportunity;  Motivation | Beliefs about capabilities;  Environmental context and resources;  Social influences;  Emotion;  Skills | Environmental restructuring;  Enablement |  | 3.1. Social support;  10.4. Social reward |
| Engaging with other attendees during and after the intervention session | - No time for networking - Anxious of making connections with others - Not seeing the value in making connections - *Provide opportunity for networking following the session within the time allocated to the session (event slot allowing)* - *Select events that incorporate networking opportunities around the session* - *Create a sense of collective action where individuals feel they are able to make a change as part of a group* | (17, 32, 49, 51-54, 56) | - Group work during the intervention session - Deliver intervention at events that provide networking opportunities in the wider programme - Provide an opportunity for individuals to introduce themselves - so people can follow-up with those with similar interests at the end of the session - Provide postcards with space to write down contacts they have made in the session | Collective efficacy – social cohesion, empowerment; Power – knowledge-based, resource-based | Opportunity;  Motivation | Reinforcement;  Social influences;  Environmental context and resources ;  Knowledge ;  Social/professional role and identity | Incentivisation;  Environmental restructuring;  Persuasion |  | 3.2. Social support (practical) |
| **Key behaviour: fostering discussion and problem solving between policy makers and the target group** | | | | | | | | | |
| Run event to establish cross-sector discussion, collaboration, networking and problem-solving | - Challenges finding a date they can all attend - Finding a location that is easy for them to attend - Making it appealing to attend - Cost to put on a good event - Time to pull the event together - *Provide content at the event that is appealing to both groups* - *Hold at a location that is appealing and easy to access for both parties e.g. London* - *Collaborate with others who are running an event - have the resources and time to organise e.g. national government Team in TRUUD, or TCAP* | Decision-makers in urban development are very busy and have multiple competing priorities that limit the time that they spend engaging in integrating health into their projects  (36)  Qualitative data from the baseline interviews - private sector actors feel that policy needs to change to support the industry to prioritise health and have some concrete suggestions on what needs to change. | - Opportunities for discussion between policy makers and private sector actors to discuss barriers and facilitators to prioritising health in the urban environment in cross-sectoral sessions (*Discourse*) - Provide link to resources they can access beyond the event - Opportunity for sharing of contacts so discussion can continue past the event. e.g. continued email correspondence - additional planned events. Names of attendees included in the programme - We will highlight this as an opportunity for private sector to have their needs heard and troubleshoot issues they may encounter. Policy makers will be able to hear directly from senior decision-makers in the private sector about what challenges they face and how policy changes could help.   The national government intervention team included:   - Postcards provided for people to write actions they will take - these will be posted 6 months later with the offer of support from the TRUUD team to help them achieve these goals | Collective efficacy – social cohesion, empowerment; Power – resource-based, knowledge-based | Capability -knowledge;  Opportunity- psychological &physical; Motivation - reflective& automatic | Knowledge;  Skills;  Social/professional role and identity;  Beliefs about capabilities;  Optimism;  Beliefs about consequences;  Goals;  Memory, attention and decision processes;  Social influences;  Emotion | Education;  Persuasion;  Incentivisation;  Coercion;  Training;  Environmental structuring;  Modelling;  Enablement |  | 1.2 Problem solving;  1.3 Goal setting (outcome)  1.6 Discrepancy between current behaviour and goal;  3.1 Social support (unspecified);  4.1 Instructions on how to perform behaviour;  5.2 Salience of consequences;  5.3 Information about environmental and social consequences;  6.1. Demonstration of the  Behavior;  6.2. Social comparison;  9.1. Credible source;  10.4. Social reward;  10.5. Social incentive;  10.7. Self-incentive;  10.9. Self-reward;  12.2. Restructuring the social  Environment;  13.2. Framing/reframing;  13.4. Valued self-identify;  13.5. Identity associated with changed  Behavior;  15.1. Verbal persuasion about  Capability; |
| **Key behaviour: target groups engaging with the intervention follow-up materials and information** | | | | | | | | | |
| Engaging in longer term networking/ discussion | - Not being aware of available networks - Not seeing the value of joining networks - *Making connections during the intervention events* - *Having an awareness of the value of the networks e.g. for answering questions about integrating health into urban development* - *Signposting to existing networks* |  | - Highlight existing networks they can join that focus on health in the urban environment - Provide an easy way for people to find out about the networks on the intervention resources webpage – QR code provided on the final slides and on the postcards on the table - On the webpage - highlight what they can expect from the network e.g. keep up to date on latest resources, tools, examples of how to integrate health into urban development/ provide a place to go to ask questions. | Collective efficacy – social cohesion, empowerment; Power – knowledge-based, resource-based | Opportunity (physical and social);  Motivation (conscious) | Knowledge;  Reinforcement;  Social influences;  Environmental context and resources | Education;  Incentivisation;  Environmental restructuring |  | 1.9. Commitment  3.2. Social support (practical)  10.5. Social incentive |
| Engagement with the intervention website/follow-up materials following the session | - Not knowing it exists - Not seeing value in it - Not having strong digital skills - *Website/newsletters contains information that the target groups have said they are interested in, tools to support prioritising issues, understanding orders of magnitude and communicating value* - *Provide information about the webpage and easy ways to access it* | (35, 36, 50, 51, 56-58). | Highlight the information contained on the website - e.g. more detailed information than was possible to cover in the intervention session, including:   - Videos of the lived experience of living in unhealthy environments - Examples of where organisations have integrated health into urban development projects - Tools that can support cost/benefit analysis of integrating health into urban development   Signpost the attendees of the intervention event to the website/other materials during the session and after if possible. Include post cards with a QR code linking to the website & include QR code on the presentation  Ask for feedback on the website to see if any more information needs to be added in follow up interviews  Make the website easy to navigate.  Ensure the website has an address that is easy to find e.g. using search engines and by typing (not too long/complicated) | Proximity – cognitive, emotional; | Opportunity (physical);  Motivation (reflective) | Knowledge;  Skills;  Reinforcement;  Intentions;  Goals;  Memory, attention and decision processes;  Social influences;  Emotion | Education;  Incentivisation;  Enablement |  | 1.9 Commitment;  4.1. Instruction on how to perform the behaviour;  10.8 Incentive (outcome) |
| **Key behaviour: engagement with research data collection** | | | | | | | | | |
| Complete surveys | - Time - Not understanding the importance - Not easy to complete - Forgetting to complete - Not liking engaging in research - *Highlight the importance of completing the survey* - *Make them as short as possible* | (17, 50) | *General*   - Explain why it is important that we collect survey data - Collect minimum data possible/make it as short as possible - Collect essential data (primary outcomes) first   *Survey on the day:*   - Provide opportunity to complete the survey on the day on paper (in-person events) - seeing others completing the surveys may normalise it for the group - Ask the industry partner to highlight how important it is to complete - TRUUD team bring attention to the survey - Have a slide on the screen highlighting the need to complete the survey   3-month follow-up surveys:   - Send via email with prompts for those who have not completed the survey. | N/A | Opportunity (physical);  Motivation (reflective) | Knowledge;  Environmental context and resources;  Social influences;  Memory, attention and decision processes | Education;  Persuasion;  Environmental restructuring;  Enablement |  | 3.1. Social support (unspecified);  4.1 Instruction on how to perform the behaviour;  6.2 Social comparison;  10.2 Material reward (behaviour);  12.1 Restructuring of the physical environment |
| Agree to be interviewed following the intervention session | - No time - Not understanding the purpose - Not interested - *Making it easy to take part, by working flexibly around their schedule* - *Provide information about how valuable it is to have their time and insights* - *Keeping the length of the interview as short as possible* | - Primary qualitative work and feedback from conversations with potential industry partners and from members of the team who work in urban development - indicated decision-makers are very busy people with lots of competing priorities (17). | -Conducting interviews to fit around the participant’s schedule  -Keeping interviews as short as possible  - Making the purpose of the interviews clear e.g. they can improve and shape the intervention and future iterations can include more of what they are interested in  - Offering phone and videocall interviews | N/A | Opportunity (physical);  Motivation (reflective) | Knowledge;  Social influences;  Social/professional role and identity;  Memory, attention and decision processes | Persuasion; |  | 10.8. Incentive (outcome);  13.4 Valued self-identity (e.g. someone who helps); |

Table 5: Coding framework for the table of changes proposed by Yardley et al.

| **Code** | **Stands For** | **Means** |
| --- | --- | --- |
| IMP | Important for behaviour change | Important change. For example, it may be important because it is likely to impact engagement or behaviour change, or for practical, ethical or safety reasons. |
| EAS | Easy and uncontroversial | An easy and feasible change that doesn’t involve any major design changes. For example, a participant was unsure of a technical term, so you add a definition. |
| REP | Repeatedly | This was said repeatedly, by more than one participant. |
| EXP | Experience | This is supported by experience. Please specify what kind of experience, for example:   1. PPIs agree this would be an appropriate change. 2. Experts (e.g. clinicians on your development team) agree that this would be an appropriate change. 3. Literature: This is supported by evidence in the literature. |
| NCON | Does not contradict | This does not contradict experience (e.g. evidence), or the Logic Model, or the Guiding Principles |
| NC | Not changed | It was decided not to make this change. Please explain why (e.g. it would not be feasible). |


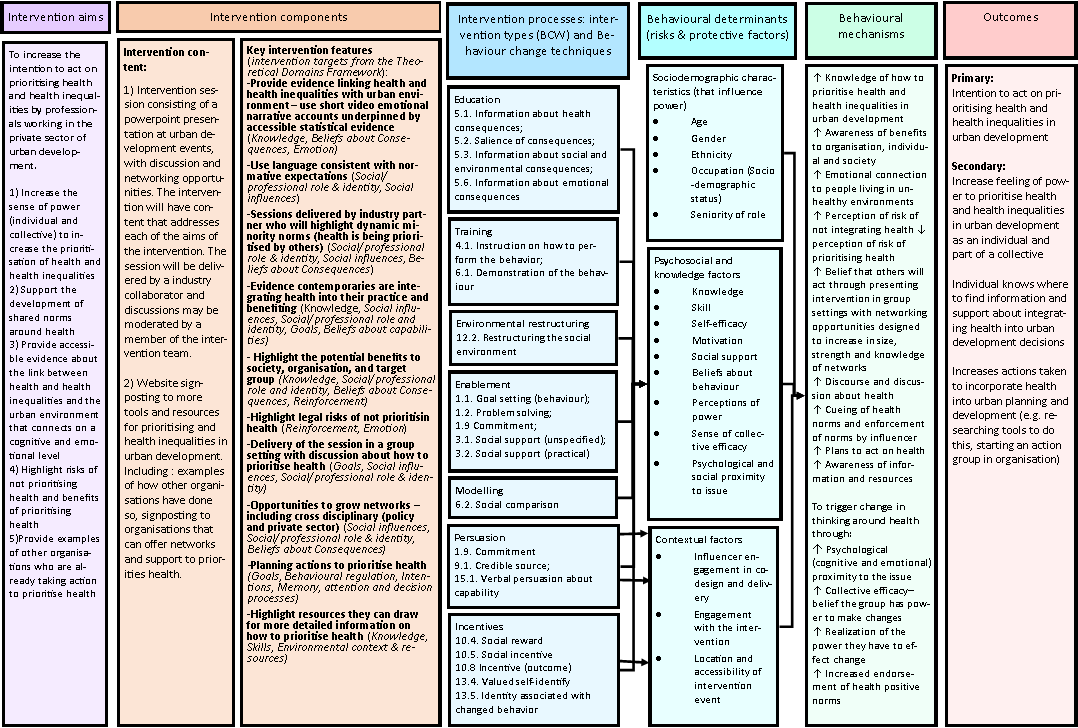

Figure 3: Full logic model
